# Comparing Care Pathways Between COVID-19 Pandemic Waves Using Electronic Health Records: A Process Mining Case Study

**DOI:** 10.1007/s41666-024-00181-6

**Published:** 2024-12-23

**Authors:** Konstantin Georgiev, Jacques D. Fleuriot, Petros Papapanagiotou, Joanne McPeake, Susan D. Shenkin, Atul Anand

**Affiliations:** 1 BHF Centre for Cardiovascular Science, Chancellor’s Building, University of Edinburgh, Edinburgh, EH16 4TJ UK; 2 Artificial Intelligence and Its Applications Institute, School of Informatics, University of Edinburgh, Edinburgh, EH8 9BT UK; 3 Independent Researcher, Edinburgh, UK; 4 The Healthcare Improvement Studies Institute, Department of Public Health and Primary Care, University of Cambridge, Cambridge, CB1 8RN UK; 5 Ageing and Health Research Group and Advanced Care Research Centre, Usher Institute, University of Edinburgh, Edinburgh, EH16 4UX UK

**Keywords:** COVID-19, Process mining, Electronic health records, Care pathways, Conformance checking, Health services

## Abstract

The COVID-19 pandemic caused rapid shifts in the workflow of many health services, but evidence of how this affected multidisciplinary care settings is limited. In this data study, we propose a process mining approach that utilises timestamped data from electronic health records to compare care provider patterns across pandemic waves. To investigate healthcare patterns during the pandemic, we collected routine events from Scottish hospital episodes in adults with COVID-19 status, generating treatment logs based on care provider input. Conformance checking metrics were used to select the Inductive Miner infrequent (IMi) algorithm for downstream analysis. Visual diagrams from the discovered Petri Nets indicated interactions on provider- and activity-level data subsets. Measures of “cross-log conformance checking” and graph edit distance (GED) further quantified variation in care complexity in adverse subgroups. Our baseline cohort included 1153 patients with COVID-19 linked to 55,212 relevant care provider events. At the conformance checking stage, the IMi model achieved good log fitness ($\overline{LF }$ $=0.95$) and generalisation ($\overline{G }$ $=0.89$), but limited precision ($\overline{PR }$ $=0.27$) across all granularity levels. More structured care procedures were present in Wave 1, compared to limited multidisciplinary involvement in Wave 2. Care activities differed in patients with extended stay ($GED=348, \overline{ PR }$ $=0.231$ *vs* $GED=197, \overline{PR }$ $=0.429$ in shorter stays). We demonstrated that process mining can be incorporated to investigate differential complexity in patients with COVID-19 and derive fine-grained evidence on shifts in healthcare practice. Future process-driven studies could use clinical oversight to understand operational adherence and driving factors behind service changes during pressured periods.

**Supplementary Information:**

The online version contains supplementary material available at 10.1007/s41666-024-00181-6.

## Introduction

The assessment, diagnosis, and management of unwell patients require the integrated work of multidisciplinary health and care teams. The increased workload, staff shortages, and the need for re-deployment during the COVID-19 pandemic caused an unprecedented reorganisation of hospital resources, particularly concerning older adults [1]. Older people recovering from severe COVID-19 conditions often require intensive rehabilitation services to regain physical function as a result of increased risk of infection and subsequent adverse outcomes [2]. The need for integrated treatment is particularly relevant for this population, as older patients often experience deconditioning after acute illness and may have a range of complex co-existing physical and mental conditions.

While striving to provide more efficient treatment for a COVID-19-affected population, the transition from Wave 1 to Wave 2 of the global pandemic also resulted in notable shifts in health and care services. Wave 2 was associated with a reduced risk of adverse outcomes in the hospital, including shorter stays and lower mortality risk [3]. In more complex cases, such as patients in intensive care units (ICU), there was a reduction of frequency in physiotherapy services, which had a negative effect on the time to first mobilisation and increased the need for continued rehabilitation after discharge [4]. The public health policy landscape rapidly changed with the introduction of shielding measures, access to testing sites and the vaccination rollout, and operational changes for the delivery of primary care. There is still surprisingly little description of changes in the interaction patterns of health and care providers for patients hospitalised with COVID-19 between Waves 1 and 2. More evidence is required to quantify these differences and establish a baseline for improvement of access to health services in the event of a future pandemic.

The increasing use of electronic health records (EHR) across the UK and abroad, integrated into modern healthcare information systems, has allowed the routine collection and analysis of detailed episodes of care, including recorded activity associated with nursing and rehabilitation staff. The use of big data has also progressed the development of tools for population-level analytics in COVID-19 patients [5]. Process mining (PM) techniques utilise timestamped event data and provide solutions that can observe “real-world behaviour” and visualise it in a manner that is understandable by non-specialists [6, 7]. A process model in the healthcare domain can be treated as a graphical representation of a series of operations performed by multidisciplinary teams. These events can be represented using the Business Process Modelling Notation (BPMN). BPMN diagrams are end-to-end flowcharts suitable for describing the interactions within a business model, including sequences in healthcare pathways [8, 9]. Workforce planning datasets across NHS Lothian, Scotland, now record timestamped interactions between hospital care providers and patients. This enables the use of PM techniques to automatically discover and compare the differences in healthcare interaction patterns.

In this case study, we used routine EHR data to extract COVID-19 hospital episodes and linked provider events, separated between the first two pandemic waves. We used these data as the basis for a PM approach consisting of four key stages:

1. PM algorithm comparison and selection for downstream analysis through conformance checking measures.
2. Development of patient subgroups for evaluating the differences in care provision for people with adverse outcomes and increased treatment complexity.
3. Cross-log conformance checking performed across the patient subgroups, including distance measures for graph similarity between Waves 1 and 2.
4. Visual process interpretation through the generation of BPMN diagrams discovered using the selected PM algorithm.

Through the output of this analysis, we aim to answer the following:

- What are the key differences in contact patterns between Waves 1 and 2? Are they consistent across the provider-level and the activity-level?
- How prevalent are these differences in patients with complex need (e.g. older people, patients with out-of-hours provision)?
- Can we use PM to discover inherent patterns in the general population that can be linked to the underlying issues of the pandemic?
- What challenges and considerations for further development of clinically meaningful process models can we identify in the context of COVID-19?

## Methods

### Data Sources

Two primary data sources were required to develop our event log and subpopulation for the study: the COVID-19 admission episodes and the linked provider contacts data. The healthcare contacts data were initially separated into two workforce planning datasets from the TrakCare (InterSystems, MA) EHR system. The first dataset included the timestamped points of contact linked to any routine or specialised care provider (e.g. nurse or general medical doctor). The second dataset consisted of additional metadata relevant to specific rehabilitation providers (e.g. physiotherapists and dieticians), including the intervention duration (total minutes) and details of the activity performed. The former helped generate the “provider-level metadata” for our event log, while the latter was used as an extension for providing the activities of the captured individuals, where possible. Intervention coding was based on a defined list of system input options covering care provider (e.g. physiotherapist) and their activities (e.g. mobilisation). This additionally split activities into assessments and interventions which were reviewed by a clinician to remove codes not associated with direct patient activity (e.g. administration). We also excluded activities coded for fewer than ten patients in our cohort. Data were processed and extracted within the DataLoch service (Edinburgh, UK) and analysed within their Secure Data Environment (SDE).

### Data Collection and Preprocessing

Our cohort of interest consisted of first hospitalisations in adult patients (aged 18 and above) who tested positive for COVID-19. We collected patient records with acute hospital admission to one of three hospitals in Edinburgh, Scotland (NHS Lothian region), between March 2020 and March 2021. Hospital admissions were identified as COVID-19 positive when any reverse transcriptase-polymerase chain reaction (RT-PCR) test was recorded during hospital stay or within 10 days of admission from community testing. Only the first hospital admission period was included for patients meeting this criterion multiple times. For patients meeting this criterion across both Wave 1 and 2, we included only their admission episode recorded during Wave 1. The cutoff points chosen for the two waves corresponded with the state of the UK pandemic (distribution plots available in Appendix B.1):

- Wave 1 (between 1st March 2020 and 31st May 2020)
- Wave 2 (between 1st September 2020 and 31st March 2021)

We restricted our cohort to having at least two contacts with at least two different care provider types recorded in our dataset to limit the population to those truly admitted for ongoing care in hospital, rather than very brief attendances for assessment. We additionally excluded cases with in-hospital death, to focus on complete patterns of care in survivors to hospital discharge. To obtain a focused view of “inter-disciplinary relationships”, we excluded self-loops present in the event log, that is, any repeating consecutive entries with the same provider over time. To avoid overlapping cases between Waves 1 and 2, we limited patient follow-up in the hospital to 90 days from admission. These steps ensured that we collected hospital events representative of urgent and routine care across multiple disciplines required for recovery to a sufficient level. Emergency department medical and nursing contacts before hospital ward admission were not captured electronically, and this element of early care could not be included in the analysis.

For our subgroup analysis, we identified six categories consisting of patients with complex care needs and adverse outcomes:

-  > 75 years old (population median across both waves)
- Admitted to ICU
- Multimorbidity (presence of two or more long-term conditions at admission)
- 1-year all-cause mortality (from the point of discharge)
- Extended hospital stay (> 30 days)
- High out-of-hours care contacts (≥ 80th percentile of patients over both waves)

We defined out-of-hours care as any contact timestamped between 7 PM and 8 AM, regardless of the day of the week. The listed baseline characteristics included age, sex, and the Scottish Index of Multiple Deprivation (SIMD), representing a relative estimate of socioeconomic status summarised through deprivation quintiles [10]. The presence of long-term conditions was extracted through national administrative records (Scottish Morbidity Record 01) covering prior hospitalisation records with relevant ICD-10 coding, using code lists defined previously by the CALIBER research group [11]. In our case, the complete list of previous conditions consisted of ischaemic heart disease, myocardial infarction, stroke, diabetes, obesity, dementia, delirium, depression, asthma, and chronic obstructive pulmonary disease (COPD).

### Process Mining Algorithms and Definitions

To describe how our PM approach captures the differences in the underlying care patterns in Waves 1 and 2, we must first define the representation of our event logs. Let $L$ denote a standardised event log over the complete population, represented by a multiset of traces $\{{t}_{1}, {t}_{2},\dots , {t}_{m}\}\in T$. Each trace ${t}_{i}$ describes a sequence of cases with an allocated timestamp from the care provider data. The number of cases captured by ${t}_{i}$ depends on the desired level of analysis and can be in the range $[2, {n}_{L}]$, where ${n}_{L}$ is the maximum amount of timestamped events for an individual patient. Process modelling is typically associated with discovering graph representations of $L$, consisting of a series of interactions between nodes ($N$) and edges ($E$). Depending on the data structure, each edge may also include a frequency weight proportional to the prevalence of a sequence in the event log, as commonly adopted in Directly-Follows Graphs (DFGs).

The most common way to express a process model is through a Petri Net representation, applied in all described PM techniques. Petri Nets are directed bipartite graphs that can be characterised through node transitions between places (individual states identified from the event log), also known as arcs. A Petri Net $P$ can be expressed as a tuple $\left(x,y, z, {m}_{0}\right)$, where $x$ and $y$ are, respectively, the sets of places and transitions describing the care patterns data, $z\subseteq \left(x\times y\right)\cup \left(y\times x\right)$ is the set of flow relations (or the set of graph arcs), and ${m}_{0}$ is the initial marking of $P$. The initial marking is an input configuration defined by assigning a number of input “tokens” to a respective place. This allows observation of concurrent executions in the Petri Net and identifies place reachability. Let us explore how the transitions of a patient requiring multidisciplinary care are expressed using this notation.

**Fig. 1:**
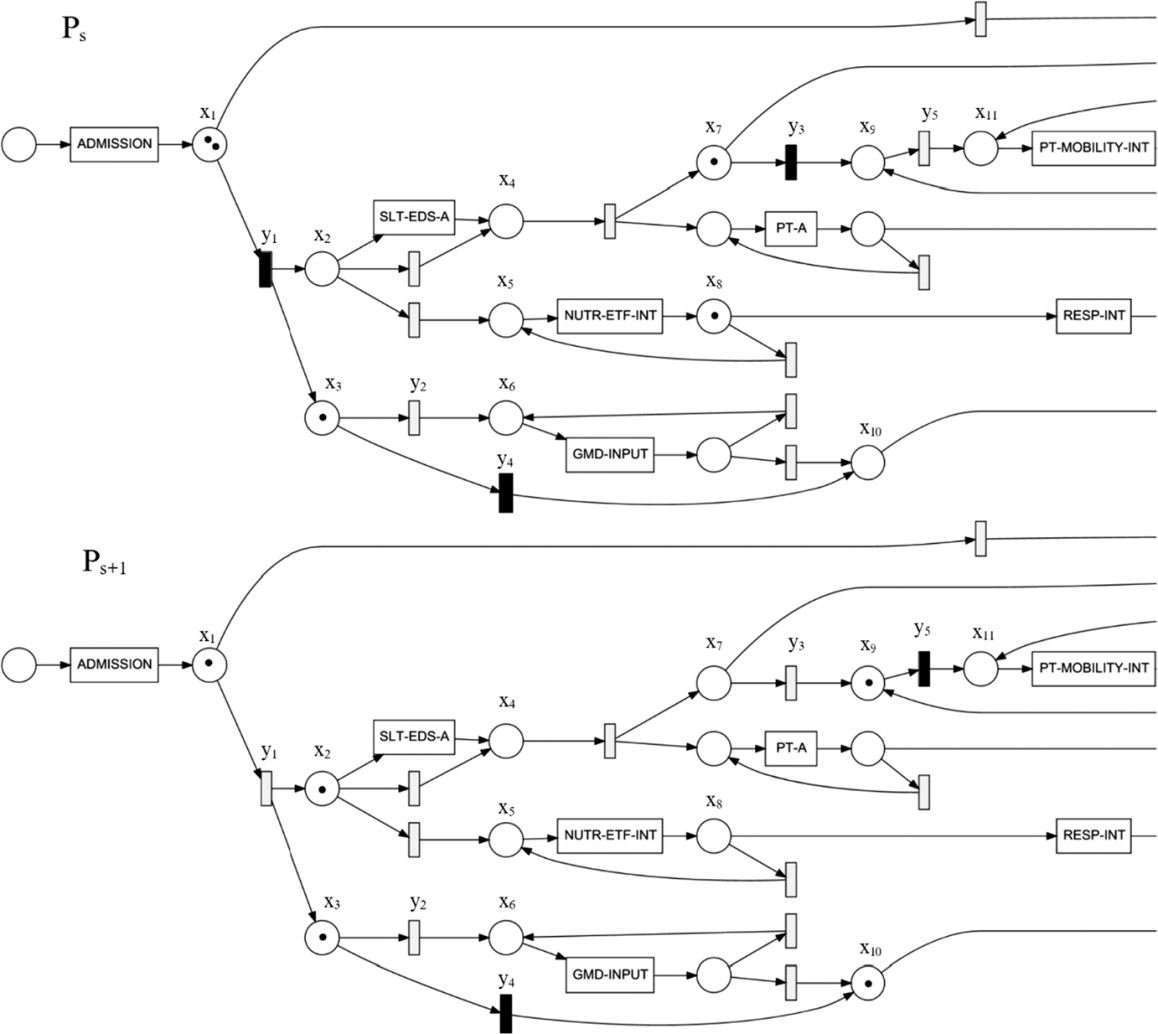
Partition of a Petri Net describing a process flow for a single patient case at an intermediate sequence ${P}_{s}$ and its follow-up markings at ${P}_{s+1}$. The enabled markings are highlighted by the respective tokens in each state (black dots) and the set of enabled transitions (black rectangles). The definitions of the coded activities are: Speech and Language Therapy – Eating, Drinking and Swallow assessment (SLT-EDS-A), physiotherapy assessment (PT-A), Physiotherapy – Mobility intervention (PT-MOBILITY-INT), Respiratory intervention (RESP-INT), Nutritional support – Enteral Tube Feeding (NUTR-ETF-INT), General Medical Doctor visit (GMD-INPUT)

We provide a visual example of this concurrent flow in Fig. 1, describing an intermediate state of the pathway ${P}_{s}$ and its follow-up sequence ${P}_{s+1}$. The initial marking of ${P}_{s}$ contains two tokens in the post-admission state (${x}_{1}$), one token prior to the split for mobility intervention (${x}_{7}$), one token after enteral tube feeding (nutritional support) intervention (${x}_{8}$), and one token prior to the split for general medical doctor visits (${x}_{3}$). At this stage, there are three enabled transitions in this part of the pathway: one post-admission (${y}_{1}$), one skipping initial general medical doctor input (${y}_{4}$), and one prior to the next transition for mobility intervention (${y}_{3}$). In this example, we assume that each enabled transition ${y}_{i}$ only consumes or produces one token at a time. After executing ${P}_{s}\to {P}_{s+1}$, these transitions are “fired” concurrently:

- ${y}_{1}$ consumes a token from ${x}_{1}$ at the “post-admission state” and produces two tokens: one prior to eating, drinking, and swallowing assessment at ${x}_{2}$ and one at ${x}_{3}$.
- ${y}_{4}$ is enabled, so it consumes a token from ${x}_{3}$ and produces one at ${x}_{10}$, skipping the general medical doctor nodes.
- ${y}_{3}$ consumes the token from ${x}_{7}$ and produces a token at ${x}_{9}$, prior to mobility intervention.
- The transition leading back to enteral tube feeding is disabled; therefore, ${x}_{8}$ must proceed forward, and emergency respiratory treatment is performed.

The remaining tokens can now be used to fire the enabled transitions ${y}_{4}$ and ${y}_{5}$ in the sequence ${P}_{s+1}\to {P}_{s+2}$. In these following events, ${y}_{4}$ will consume the token from ${x}_{3}$ and produce a token in ${x}_{10}$ and ${y}_{5}$ will consume a token from ${x}_{9}$ and produce a token in ${x}_{11}$ leading up to a mobility-related physiotherapy treatment. Additionally, at the current step, there are no enabled transitions after ${x}_{2}$, which indicates that an assessment for eating, drinking, and swallowing problems is executed, transferring this token over to ${x}_{4}$. Thus, the Petri Net captures these transitions in care as a dynamic system of discrete events, where any deviation from the original event log will be reflected during execution (e.g. missing tokens during an enabled transition). The BPMN diagram is a simplified version of a Petri Net that does not include transitional states but describes the overall process flow through decision nodes: AND-split, AND-join, XOR-split, and XOR-join. These nodes indicate the discovered constraints by the process model for the respective granularity level.

In our baseline performance comparison, we evaluated four process discovery algorithms: Alpha Miner (AM), Inductive Miner infrequent (IMi), Inductive Directly-Follows Miner (IDFG), and Heuristics Miner (HM). A summary of each of these algorithms and their ways of discovering a Petri Net from an input event log is given in Appendix A.1. The noise threshold $\theta \in [\text{0,1}]$ present in IMi, IDFG, and HM (lower value indicates a stronger adjustment) is used to reduce model complexity and over-approximation of decision nodes that can result in intractable paths within the Petri Net. In the context of HM, $\theta$ is used to prune weak causal dependencies (eventually follows relations) in the model.

### Conformance Checking for Performance and Subgroup Analysis

Similarly to the example shown in Fig. 1, we use a technique known as “token-based replay” [12] to measure conformance between a Petri Net and an event log or similarity between two discovered Petri Nets. After executing the sequences across the enabled transitions in the Petri Net, a set of four values (${n}_{m}, {n}_{c}, {n}_{p}, {n}_{r})\in {t}_{i}$ is computed for each trace, describing the number of missing, consumed, produced, and remaining tokens, respectively. These values are used to estimate the cumulative log fitness ($LF\in [\text{0,1}]$), where a higher value means the final marking has been reached with less remaining and missing tokens from the Petri Net. Other conformance measures include precision ($PR\in \left[\text{0,1}\right]$), [13] which looks at the accuracy of follow-up transitions for each marking and generalisation ($G\in \left[\text{0,1}\right]$), [14] which measures the average transition frequency compared to the original event log. Some well-known problems of token-based replay as an algorithm include token flooding (over-population of missing tokens for vastly different cases) and long transitions due to many identified fall-throughs severely affecting computational speed. However, we use an improved version that identifies the local shortest paths between places with enabled transitions [15]. This localisation technique allows for more robust diagnostics in model deviations when performing token-based replay. Additionally, we use the graph edit distance [16] ($GED\in [1, \infty ))$ as a quantitative measure of similarity between two Petri Nets (${P}_{i}, {P}_{j}$), describing the minimum number of edit operations (insertion, deletion, or substitution of nodes/edges) required to transform ${P}_{i}$ into ${P}_{j}$. By default, each edit operation is assigned a cost of 1, which is accumulated upon traversing the graphs.

In the context of this case study, we use ${L}_{1}$ and ${L}_{2}$ to denote the sub-logs representing Waves 1 and 2 and ${P}_{1}$ and ${P}_{2}$ to denote the discovered Petri Nets of these sub-logs, respectively. When we perform performance comparison, we use token-based replay on a Petri Net against its original event log, i.e. we estimate the log fitness ($LF\left({P}_{i}\right)$), precision ($PR\left({P}_{i}\right)$), and generalisation ($G\left({P}_{i}\right)$) per cohort. When we perform subgroup analysis, we replay the Petri Nets against their counterpart event logs, i.e. we estimate the “cross-log fitness” ($LF({L}_{i}, {P}_{j})$), precision ($PR({L}_{i}, {P}_{j})$), and generalisation ($G({L}_{i}, {P}_{j})$). Further details regarding the process model definitions and estimation of the conformance checking measures are provided in Appendix A.2.

### Implementation Tools and Software

The primary PM tools used for generating the analysis in this case study include *bupaR*, *pm4py*, and *ProM*. All preliminary data extraction, preprocessing, and process model generation were performed within the SDE using R (version 4.2.0). We used the base *bupaR* framework [17] (version 0.5.2) to generate the target event log, produce summary statistics, and generate the DFGs used in the IDFG models. Then, to obtain the Petri Nets (except for IDFG) and evaluate them using token-based replay, we used the *R interface to the pm4py library* [18] (version 1.2.7). We imported the generated Petri Net markings in Petri Net Markup Language (PNML) format to *ProM* [19] (version 6.13) and converted them into BPMN diagrams for visual analysis using the function “Convert Petri net to BPMN diagram”.

Additionally, we used *ProM* to discover the IDFG Petri Nets from the acquired DFGs. This was done using the function “Mine Petri net with Inductive Miner – directly-follows”. Afterwards, the IDFG Petri Nets were evaluated using the same approach but on the standard *pm4py* library (version 2.7, Python 3.10). To generate the GEDs between two input Petri Nets, we used the *NetworkX* (version 3.2) package in Python with a fixed timeout limit of 30 s.

### Overview of the Approach

**Fig. 2:**
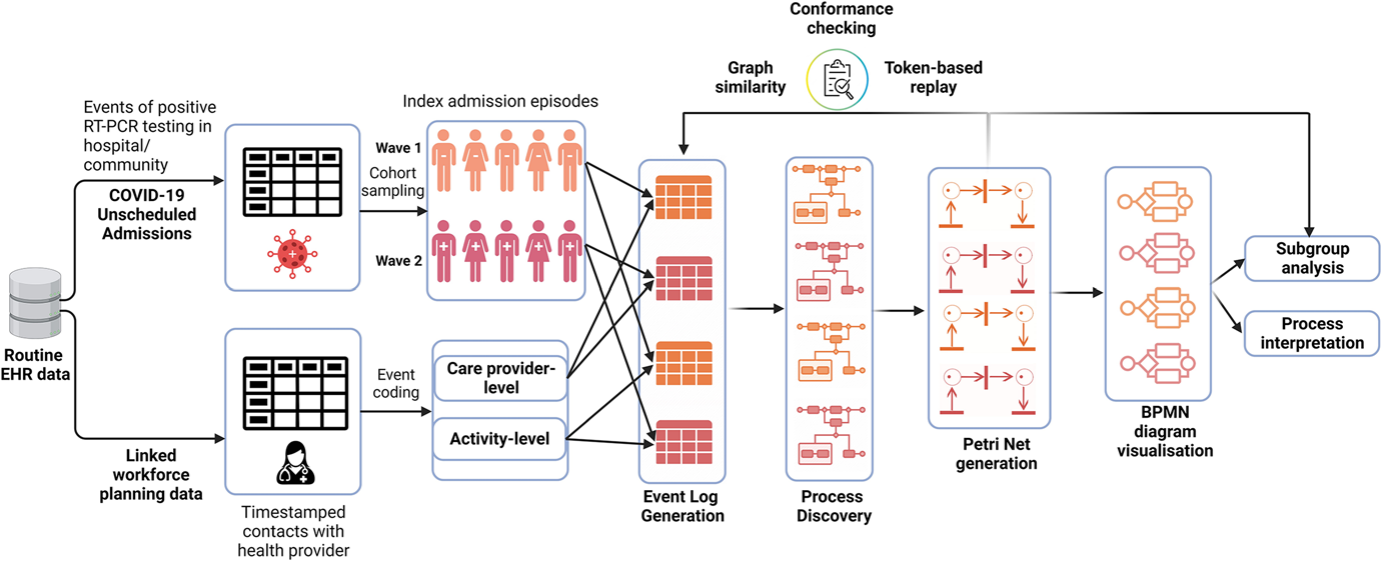
Conceptual flowchart describing the steps performed to transform the routinely collected data for process modelling and downstream analysis of the two COVID-19 cohorts

Now, we can review our overall pipeline and the steps required to produce the performance and subgroup analysis for this case study. The concept flowchart in Fig. 2 highlights these steps. We first determine the positive COVID-19 episodes linked to the EHR data and use the described metadata to extract patient outcomes and stratify the population into cohorts representing Waves 1 and 2.

We used the care provider contacts data to code and aggregate provider and activity-level events, which allowed us to generate four event log variants across the two waves. The next step involved process discovery, where each model type was evaluated using token-based replay on the generated Petri Net against its original event log. Then, the best-performing process model was selected for subgroup analysis. We additionally converted the Petri Nets to BPMN diagrams to visually compare the quality of the discovered process models.

## Results

### Patient and Event Log Summary Data

In our final population, we included 1153 patients presenting with COVID-19, with 302 (26%) in Wave 1 and 851 (74%) in Wave 2. The baseline characteristics and outcomes for the two cohorts are presented in Table 1. We can observe that the extracted episodes of care detailed predominantly older patients (76 [62, 85] years). People accessing healthcare services in Wave 1 were based in less deprived regions in Scotland (*p* = 0.003 across the SIMD category). We measured the differences across some of the most common admitting specialties, with respiratory care being more prevalent in Wave 1 (9% vs 2% in Wave 2). Among the reported outcomes, only death at 1-year was significantly different (19% in Wave 2 vs 13% in Wave 1, *p* = 0.018). Patients in Wave 1 received a higher number of “rehabilitation-specific sessions”, with 19 (6, 48) vs 15 (6, 35) contacts in Wave 2, *p* = 0.011 across 410 (130, 935) vs 292 (110, 710) rehabilitation minutes in Wave 2, *p* = 0.02.

**Table 1:** Baseline characteristics describing the two COVID-19 cohorts

Characteristic	All (*n* = 1153)	Wave 1 (*n* = 302)	Wave 2 (*n* = 851)	*p*
Age (median, IQR)	76 (62, 85)	75 (60, 84)	76 (63, 85)	0.15
Sex				0.185
Male	554 (48%)	155 (51%)	399 (47%)	
Female	599 (52%)	147 (49%)	452 (53%)	
SIMD in quintiles				0.003
1 (most deprived)	158 (14%)	29 (10%)	129 (15%)	
2	325 (28%)	69 (23%)	256 (30%)	
3	178 (15%)	50 (17%)	128 (15%)	
4	200 (17%)	64 (21%)	136 (16%)	
5 (least deprived)	292 (25%)	90 (30%)	202 (24%)	
Admission specialty				< 0.001
General Medicine	753 (65%)	195 (65%)	558 (66%)	
Intensive Therapy	75 (7%)	24 (8%)	51 (6%)	
Orthopaedics	79 (7%)	17 (6%)	62 (7%)	
Respiratory Medicine	40 (4%)	26 (9%)	14 (2%)	
Other	206 (18%)	40 (13%)	166 (20%)	
Outcomes				
Multimorbidity	374 (32%)	104 (34%)	270 (32%)	0.387
Length of stay, days (median, IQR)	25 (12, 50)	21 (11, 47)	26 (13, 51)	0.093
Extended stay (> 30 days)	498 (43%)	125 (41%)	373 (44%)	0.462
Death (1-year all-cause mortality)	204 (18%)	40 (13%)	164 (19%)	0.018
Provider summary				
Total number of contacts (median, IQR)	64 (31, 135)	66 (30, 155)	63 (31, 131)	0.496
Out-of-hours contacts (median, IQR)	19 (10, 38)	18 (10, 38)	20 (10, 39)	0.588
Rehabilitation needs				
Total number of rehabilitation contacts (median, IQR)	16 (6, 39)	19 (6, 48)	15 (6, 35)	0.011
Total minutes of rehabilitation (median, IQR)*	325 (110, 760)	410 (130, 935)	292 (110, 710)	0.02
Days to first rehabilitation contact (median, IQR)	3 (2, 5)	3 (2, 5)	3 (1, 5)	0.034
Days to second rehabilitation contact (median, IQR)	4 (2, 6)	4 (2, 6)	3 (2, 6)	0.024

Values are in proportion of patients (%) unless stated otherwise

*SIMD* Scottish Index of Multiple Deprivation

Out-of-hours is defined as any contact with a health provider between 7 AM and 8 PM

Significance was measured between Wave 1 and 2 groups using the chi-squared test for categorical variables and the Mann–Whitney *U* test for non-normally distributed continuous variables. *P*-values were not adjusted for multiple testing

The asterisk symbol (*) excludes missing values in 38.5% of patients

Our complete standardised event log consisted of 55,212 events on both the activity and provider levels (15,954 in Wave 1 and 37,912 in Wave 2). The breakdown of each coded care provider and defined activity can be observed in Table 2. Overall, we collected 11 unique provider types and 23 related activities, which were present in both waves. Unsurprisingly, the most common activity type involved standard nursing care services. The most prevalent specialist care was related to physiotherapy and occupational therapy. This set was substantially more limited as 14% of events were not identified due to missing data (labelled OTHER). A further 12% (REHAB-ANY-INT) were identified from the rehabilitation dataset but did not contain a sufficient description. Additionally, another 1% of the treatments (TRTFAILED) were not completed because the patient was too unwell or declined a rehabilitation session. Despite these limitations, the activity set provided more detailed dependencies for rehabilitation care provision. Similar to the length of stay, we also observed a slightly higher overall case duration per patient in Wave 2 (26 days vs 21 days in Wave 1).

**Table 2:** Summary of coded events and event log metadata

Event description		Event code	Overall	Wave 1	Wave 2
Start/end points	Point of admission	ADMISSION	2094	302	851
	Point of discharge	DISCHARGE	1419	274	740
Provider-level	Standard nursing care provider	NURSE	18,706	5160	13,546
	Physiotherapist	PT	14,056	4150	9906
	Occupational therapist	OT	6506	1866	4640
	General medical doctor	GMD	6158	2185	3973
	Dietician	DT	3298	1157	2141
	Other type of provider	OTHER	1146	354	792
	Clinical pharmacist	CP	657	192	465
	Speech and language therapist	SLT	635	232	403
	Palliative care provider	PC	297	12	285
	Specialised nursing care provider	SP_NURSE	157	63	94
	Support worker	SUPPW	83	7	76
Activity-level	Standard nursing input	ST-NURSING	18,706	5160	13,546
	Other types of care provision	OTHER-INPUT	7822	2340	5482
	Any “rehabilitation-related intervention”	REHAB-ANY-INT	6360	1815	4545
	General medical doctor input	GMD-INPUT	6158	2185	3973
	Any physiotherapy intervention	PT-ANY-INT	5439	1484	3955
	Occupational therapy assessment	OT-A	1576	513	1063
	Physiotherapy assessment	PT-A	1170	397	773
	Clinical pharmacist input	CP-INPUT	657	192	465
	Oral Nutritional Supplements (ONS) intake	NUTR-ONS-INT	621	215	406
	Treatment failed (patient too unwell, refused access, and/or unsuitable to treat)	TRTFAILED	612	218	394
	Enteral tube feeding intervention	NUTR-ETF-INT	527	250	277
	Respiratory intervention	RESP-INT	527	189	338
	Palliative care input	PC-INPUT	297	12	285
	Assessment for eating, drinking, and swallowing problems	SLT-EDS-A	284	121	163
	Intervention for eating, drinking, and swallowing problems	SLT-EDS-INT	195	58	137
	Food fortification	NUTR-FF-INT	193	55	138
	Mobility training	PT-MOBILITY-INT	176	59	117
	Intervention for speech, language, and communication problems	SLT-SLC-INT	120	54	66
	Support worker input	SUPPW-INPUT	83	7	76
	Nutritional assessment	NUTR-A	65	11	54
	Assessment for speech, language and communication problems	SLT-SLC-A	43	11	32
	Mobility assessment	PT-MOBILITY-A	36	18	18
	Specialised nursing care input	SP-NURSING	32	14	18
Event log summary	Total number of events	/	55,212	15,954	37,912
	Median case duration (days)	/	24.89	21.35	25.67
	Median case duration (hours)	/	597.27	512.49	616.28

### Model Performance Comparison

Using the above-defined PM algorithms and measures of conformance checking, we applied token-based replay and computed the log fitness, precision, and generalisation of each discovered Petri Net against its original event log. We additionally tested the models with more relaxed ($\theta =0.7$) and strict ($\theta =0.99$) noise and dependency thresholds. The full results displayed in Table 3 show that, overall, there is always a tradeoff between precision and log fitness, and it is difficult to achieve good precision when the model effectively captures the complete set of states present in the event log.

**Table 3:** Model performance comparison in different process mining algorithms

Model type		$\theta$	Wave 1			Wave 2			Mean		
			$LF\left({P}_{1}\right)$	$PR\left({P}_{1}\right)$	$G\left({P}_{1}\right)$	$LF\left({P}_{2}\right)$	$PR\left({P}_{2}\right)$	$G\left({P}_{2}\right)$	$\overline{LF }$	$\overline{PR }$	$\overline{G }$
Provider-level	AM	.	0.116	0.73	0.9	0.138	0.847	0.955	0.127	0.788	0.928
	HM	0.7	0.979	0.156	0.804	0.977	0.172	0.797	0.978	0.164	0.801
	HM	0.99	0.741	0.494	0.808	0.806	0.647	0.773	0.773	0.57	0.791
	*IMi*	*0.7*	*0.982*	*0.371*	*0.917*	*0.945*	*0.188*	*0.917*	*0.964*	*0.279*	*0.917*
	IMi	0.99	1	0.139	0.843	1	0.17	0.917	1	0.155	0.88
	IDFG	0.7	0.375	0.668	0.903	0.222	0.774	0.896	0.298	0.721	0.9
	IDFG	0.99	0.285	0.664	0.892	0.326	0.758	0.911	0.306	0.711	0.902
Activity-level	AM	.	0.19	0.467	0.871	0.164	0.579	0.92	0.177	0.523	0.896
	HM	0.7	0.969	0.09	0.769	0.969	0.097	0.78	0.969	0.094	0.774
	HM	0.99	0.726	0.301	0.821	0.937	0.256	0.807	0.832	0.279	0.814
	*IMi*	*0.7*	*0.947*	*0.379*	*0.885*	*0.933*	*0.123*	*0.844*	*0.94*	*0.251*	*0.864*
	IMi	0.99	0.999	0.074	0.812	1	0.096	0.895	1	0.085	0.853
	IDFG	0.7	0.636	0.431	0.898	0.703	0.551	0.922	0.67	0.491	0.91
	IDFG	0.99	0.833	0.243	0.878	0.813	0.369	0.903	0.823	0.306	0.89

Conformance checking metrics stratified by wave: ${\varvec{L}}{\varvec{F}}{({\varvec{P}}}_{{\varvec{i}}})$, log fitness**,** ${\varvec{P}}{\varvec{R}}{({\varvec{P}}}_{{\varvec{i}}})$, precision, ${\varvec{G}}{({\varvec{P}}}_{{\varvec{i}}})$, generalisation. Noise (IMi) or Dependency (HM) threshold: ${\varvec{\theta}}$. IDFG: Inductive Miner process discovery using the generated Directly-follows Graph in bupaR, instead of a standard event log. Marked in italic is the model chosen for downstream analyses

Models, such as AM and IDFG (dependencies highlighted in Appendix B.2), capture a higher model precision but represent a smaller subset of the event log behaviour. In contrast, the IMi and HM models with strict thresholds capture almost all state transitions but achieve poor precision. Ultimately, the best model for this case is achieved by relaxing the noise threshold $\theta$ for the IMi model, limiting some of the captured behaviour ($\overline{LF }$ $=0.964$ on the provider-level and $\overline{LF }$ $=0.94$ on the activity-level) but representing a better fit for follow-up transitions ($\overline{PR }$ $=0.279$ on the provider-level and $\overline{PR }$ $=0.251$ on the activity-level). The IMi model generalisation was also the highest among models that achieved a $L{F}_{i}>0.9$. It is also worth noting that upon adding this noise threshold, precision was still relatively poor for Wave 2 (particularly on the activity level) due to higher sequencing variability than Wave 1.

### BPMN Diagrams of the Discovered Process Models

**Fig. 3:**
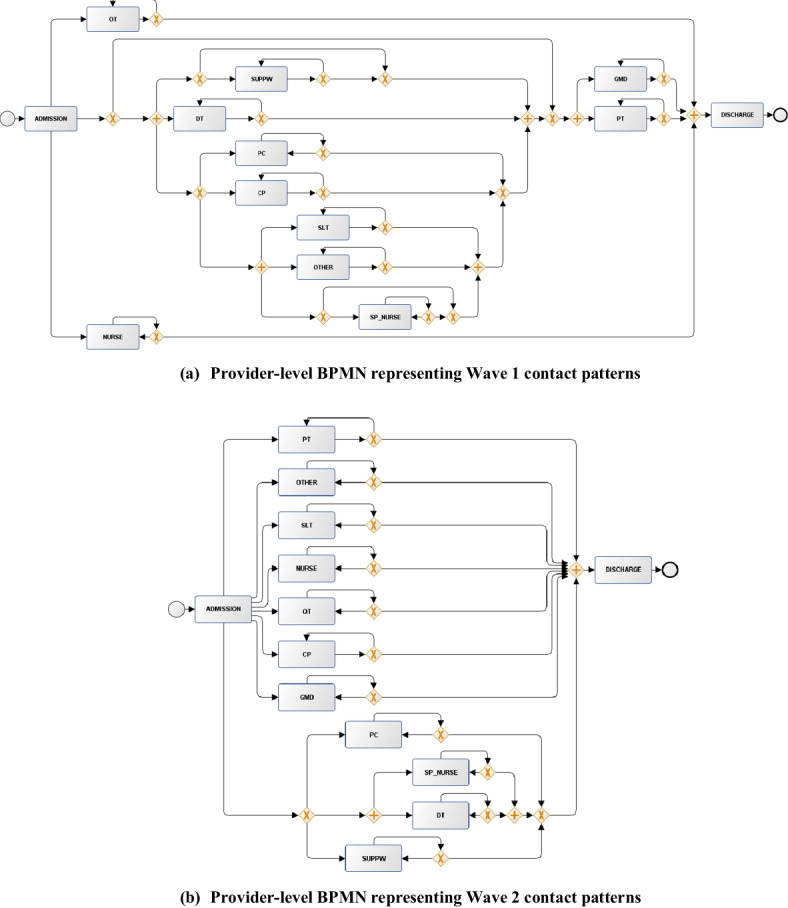
BPMN flow diagrams discovered by the IMi model on the “provider-level event logs”. Inputs generated from the discovered Petri Nets using ProM

In the following analysis, we used the output Petri Nets for each subset of the IMi model to visualise the discovered interaction patterns. We converted them to BPMN format, which highlighted the care provider dependencies through decision nodes (“ + ” nodes indicating dependent activities (AND-join, AND-split) and “X” nodes indicating exclusive choice operations (XOR-join, XOR-split)). Figure 3 shows the discovered set of provider-level dependencies. In Wave 1, nursing care (NURSE) and occupational therapy (OT) visits occurred independently, while the middle part of the diagram included multiple levels of interaction rules. For example, physiotherapy (PT) contacts were associated with multidisciplinary care from other providers including general medical doctors (GMD), speech and language therapy (SLT), and dietician support (DT). By comparison, the Wave 2 BPMN highlighted that many providers were more independently linked to discharge, with limited multidisciplinary care. The only independent “AND-split” rule discovered by the process model was linked to interactions between dieticians (DT) and specialised nurses (S_NURSE), suggesting combined care for those requiring complex feeding support [20]. The limited involvement in multidisciplinary care during Wave 2 may be explained by lower disease severity. As seen in Table 1, overall rehabilitation contacts were fewer in this cohort. This may also mean that hospital pressures in Wave 2 resulted in more focus on single-specialty-led treatment rather than multidisciplinary care.

**Fig. 4:**
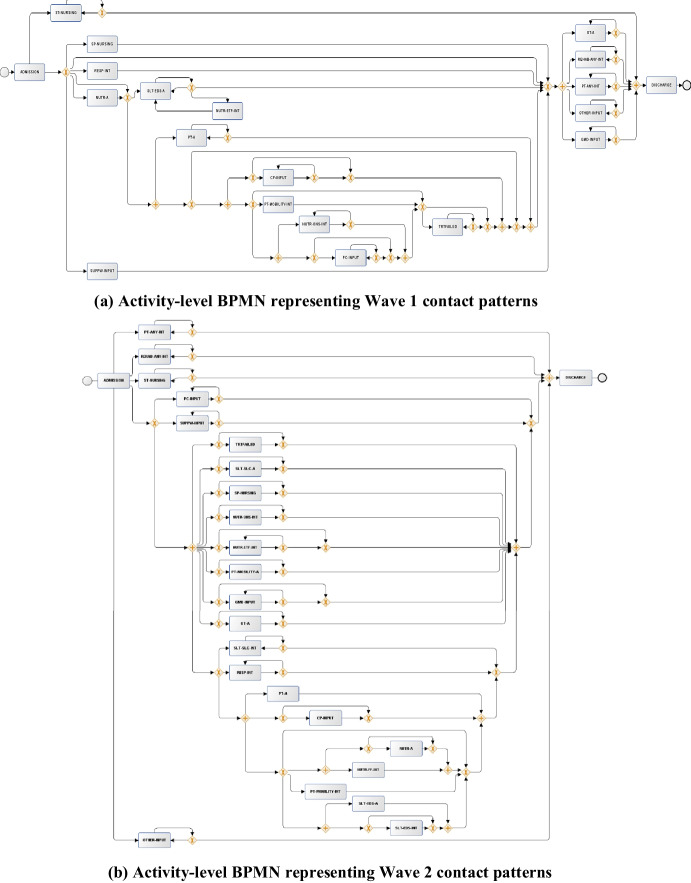
BPMN flow diagrams discovered by the IMi model on the “activity-level event logs”. Inputs generated from the discovered Petri Nets using ProM

A similar pattern is observed in the “activity-level BPMN diagrams” (Fig. 4). In Wave 1, activities such as specialist nursing care (SP-NURSING) were executed early in the pathway with downstream dependencies for further activities that followed. In contrast, in the Wave 2 BPMN, more simultaneous activity was observed with fewer dependencies. As it might be expected, associations were present between SLT and DT activities, such as interventions for eating, drinking, and swallowing (SLT-EDS-A, SLT-EDS-INT) and food fortification (NUTR-FF-INT). With the return of hospital referrals in Wave 2, it is possible that this affected the required coordination to streamline the patient journey. As referrals were not generally provided in Wave 1 and the focus was on immediate involvement in active cases, it may be less surprising that the process models discovered more structured patterns during this time.

### Comparison Between Complex Patient Subgroups

After observing the underlying care patterns through the BPMN diagrams, we now focus on the similarities of individual subgroups across waves and the differences between care provision for adverse and non-adverse cases. In this subgroup analysis, we highlighted these differences using the cross-log conformance checking method and the Petri Net GED (Table 4). Across all subgroup types, we observed relatively high log fitness ($\overline{LF }$ $>0.9$), indicating that the trace subtypes were not substantially different. Precision was relatively higher on the activity-level, where model behaviour and follow-up sequences of activities were more similar between the waves. Generalisation was slightly better when replaying the log of Wave 2 against the Petri Nets of Wave 1 ($G({L}_{2},{W}_{1})$) compared to the opposite. On the activity-level, differences between complex and less complex cases were more prevalent than at the provider-level, as indicated by the GED and the log fitness. The most remarkable differences were in pathways in older patients (according to the GED) and pathways with high out-of-hours care (according to the log fitness). Intensive therapy was more standardised between the waves. This was expected, considering this specialty contains intervention sequences and timing guidelines. Care activities were substantially different in patients with an extended stay in hospital ($GED=348, \overline{P }$ $=0.231$ in the positive group and $GED=197, \overline{P }$ $=0.429$ in the negative group). However, 1-year mortality, multimorbidity, and out-of-hours care were not associated with significant differences between groups. Overall, there were contradictions between the estimated differences in the provider and activity levels, but the activity subsets seemed to align better between the waves regarding observed behaviour. This resulted in more pronounced distances between adverse and non-adverse groups. These included increased variation in older groups and extended stay patients and decreased variation among the rest.

**Table 4:** Multi-level cross-log conformance checking comparison over each patient subgroup

Subset			Log 1 to Wave 2 model replay			Log 2 to Wave 1 model replay			Mean			$\mathbf{G}\mathbf{E}\mathbf{D}$
			${\varvec{L}}{\varvec{F}}\left({{{\varvec{L}}}_{1},{ }{\varvec{P}}}_{2}\right)$	${\varvec{P}}{\varvec{R}}\left({{{\varvec{L}}}_{1},{ }{\varvec{P}}}_{2}\right)$	${\varvec{G}}\left({{{\varvec{L}}}_{1},{ }{\varvec{P}}}_{2}\right)$	${\varvec{L}}{\varvec{F}}\left({{{\varvec{L}}}_{2},{ }{\varvec{P}}}_{1}\right)$	${\varvec{P}}{\varvec{R}}\left({{{\varvec{L}}}_{2},{ }{\varvec{P}}}_{1}\right)$	${\varvec{G}}\left({{{\varvec{L}}}_{2},{ }{\varvec{P}}}_{1}\right)$	$\overline{{\varvec{L}}{\varvec{F}} }$	$\overline{{\varvec{P}}{\varvec{R}} }$	$\overline{{\varvec{G}} }$	
Provider-level	Age	> 75	0.962	0.158	0.655	0.973	0.394	0.705	0.967	0.276	0.68	142
		≤ 75	0.965	0.136	0.76	0.971	0.442	0.761	0.968	0.289	0.76	161
	Intensive therapy	Y	0.986	0.118	0.793	0.984	0.133	0.84	0.985	0.126	0.817	120
		N	0.952	0.147	0.795	0.972	0.522	0.866	0.962	0.335	0.83	176
	Extended stay	Y	0.978	0.12	0.74	0.969	0.147	0.915	0.974	0.133	0.828	154
		N	0.923	0.195	0.696	0.977	0.516	0.881	0.95	0.356	0.788	166
	1-year mortality	Y	0.964	0.145	0.637	0.967	0.201	0.893	0.966	0.173	0.765	143
		N	0.967	0.144	0.729	0.945	0.185	0.922	0.956	0.164	0.826	134
	Multi-morbidity	Y	0.971	0.177	0.631	0.964	0.451	0.793	0.967	0.314	0.712	148
		N	0.963	0.136	0.751	0.969	0.369	0.91	0.966	0.253	0.83	148
	Out-of-hours care	Y	0.985	0.118	0.677	0.983	0.125	0.934	0.984	0.121	0.806	158
		N	0.926	0.197	0.696	0.957	0.517	0.869	0.942	0.357	0.783	176
Activity-level	Age	> 75	0.925	0.396	0.629	0.939	0.483	0.883	0.932	0.439	0.756	335
		≤ 75	0.918	0.357	0.714	0.938	0.57	0.87	0.928	0.463	0.792	141
	Intensive therapy	Y	0.971	0.333	0.58	0.979	0.439	0.691	0.975	0.386	0.636	185
		N	0.942	0.1	0.784	0.936	0.45	0.897	0.939	0.275	0.841	354
	Extended stay	Y	0.963	0.078	0.727	0.949	0.384	0.924	0.956	0.231	0.826	348
		N	0.896	0.432	0.619	0.921	0.425	0.842	0.909	0.429	0.73	197
	1-year mortality	Y	0.941	0.467	0.598	0.937	0.475	0.854	0.939	0.471	0.726	194
		N	0.937	0.098	0.749	0.935	0.43	0.9	0.936	0.264	0.825	306
	Multi-morbidity	Y	0.951	0.429	0.673	0.915	0.522	0.771	0.933	0.475	0.722	211
		N	0.942	0.277	0.686	0.941	0.458	0.881	0.942	0.367	0.783	331
	Out-of-hours care	Y	0.972	0.311	0.752	0.967	0.244	0.899	0.969	0.278	0.825	221
		N	0.921	0.392	0.608	0.908	0.432	0.89	0.915	0.412	0.749	339

Wave 1 Log conformance to the Wave 2 Petri Net is defined as follows: ${\varvec{L}}{\varvec{F}}\left({{{\varvec{L}}}_{1},\boldsymbol{ }{\varvec{P}}}_{2}\right)$ log fitness, ${\varvec{P}}\left({{{\varvec{L}}}_{1},\boldsymbol{ }{\varvec{P}}}_{2}\right)$ precision, $\mathbf{G}\left({{\mathbf{L}}_{1},\mathbf{P}}_{2}\right)$ generalisation. Wave 2 Log conformance to the Wave 1 Petri Net is defined as follows: ${\varvec{L}}{\varvec{F}}\left({{{\varvec{L}}}_{2},\boldsymbol{ }{\varvec{P}}}_{1}\right)$ log fitness, ${\varvec{P}}\left({{{\varvec{L}}}_{2},\boldsymbol{ }{\varvec{P}}}_{1}\right)$ precision, ${\varvec{G}}\left({{{\varvec{L}}}_{2},\boldsymbol{ }{\varvec{P}}}_{1}\right)$ generalisation, ${\varvec{G}}{\varvec{E}}{\varvec{D}}$ graph edit distance

**Fig. 5:**
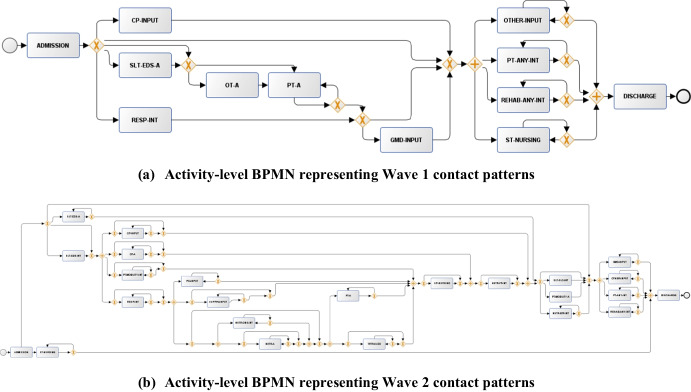
BPMN flow diagrams discovered by the IMi model on the “activity-level event logs” in patients older than 75 years

To better understand the implications of the GED scores, we visualised BPMN diagrams across subgroups with high and low variability between the pandemic waves. Regarding high variability, we focused on care pathways in patients older than 75 (Fig. 5). On the activity-level, Wave 2 pathways were clearly associated with a wider range of activities across multiple disciplines in these patients.

**Fig. 6:**
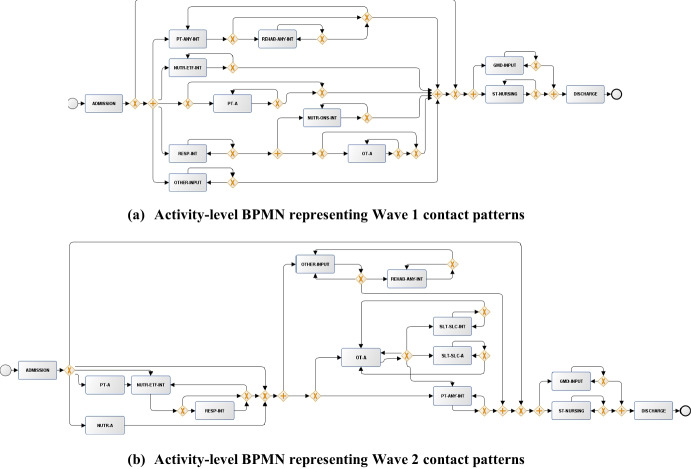
BPMN flow diagrams discovered by the IMi model on the “activity-level event logs” in patients with intensive therapy

The Wave 2 BPMN reveals more interdisciplinary relationships between nursing and rehabilitation services with possible interactions between nutrition support (NUTR-A, NUTR-ONS-INT), physiotherapy (PT-A), and palliative care providers (PC-INPUT) linked to follow-up activities of physiotherapy (PT-MOBILITY-A) and medical doctor input (GMD-INPUT). The level of multidisciplinary input for these older patients was much greater than the overall population (Fig. 4b). This may suggest that a larger portion of healthcare resources were directed towards care for older people during Wave 2. Regarding low variability, we analysed the pathways of patients in intensive therapy with similar GED scores (Fig. 6). In this case, the range of activities was similar. In both waves, routine nursing care (ST-NURSING) and medical doctor inputs (GMD-INPUT) depended on other interdisciplinary domains. Additional BPMN diagrams are provided in Appendix B.3, detailing the pathways of the remaining subgroups. Furthermore, we performed a stratified conformance checking analysis across sex and SIMD quintiles (Appendix B.4). On the activity-level, care pathways in male patients varied more than in female patients. Interestingly, substantially fewer changes in intervention patterns were detected in patients from areas with high deprivation $(GED=80)$. We additionally analysed the trace coverage rates (using the “provider-level model”) between subgroups to better understand the log fitness (Appendix B.5). These results aligned with some of the findings from the “provider-level subsets” in Table 4. The linear relationships between the conformance checking metrics and the GED were also presented.

## Discussion

Identifying and measuring care patterns throughout the COVID-19 pandemic is challenging due to the never-before-seen demand for hospital services. We proposed a comprehensive PM approach utilising EHR data to understand the variation in healthcare provider patterns between the first two pandemic waves. Through the model performance and BPMN diagrams, we observed the ability of the IMi model to identify split and join rules across disciplines and activities. At baseline, patients were similar in age and sex, but those in Wave 2 who survived hospital discharge had a significantly higher all-cause death within the following year. Patients in Wave 2 had a longer length of stay but with fewer rehabilitation sessions and associated minutes of therapy. Fewer dependencies between care providers were discovered in Wave 2, suggesting simpler care pathways than in Wave 1, except for those older than 75 years old and those with extended hospital stays, where complexity was higher in Wave 2. In a more detailed analysis, this variation was driven at the activity-level, with greater input differences from a similar range of care providers. In contrast, care provider and activity pathways for those who received high out-of-hours care and intensive therapy were similar between waves. It is possible that the reintegration of routine care alongside COVID-19 specialised care in Wave 2 introduced limited capacity in hospital systems and promoted less coordinated multidisciplinary care.

There are several strengths to our case study. Our use of routine healthcare data, including fine-grained health contacts and associated in-hospital activities, can be used to define novel quantifiable measures of differential complexity in treatment between two cohorts. We showcased that PM can enhance data-driven analytics over comparative studies through cross-log similarity measures between care pathways. Visual BPMN diagrams supported these estimates by showcasing specific changes in healthcare patterns on a fine-grained level. We selected the most suitable process model to perform process discovery through standardised conformance checking measures. The COVID-19 pandemic initiated a period of exceptional hospital pressures, resulting in novel shifts in treatment pathways as part of infection control measures. However, there is still limited understanding towards how well hospital systems adapted to these changes and how this affected acutely ill patients. Thus, we provided a detailed investigation of care coordination during the pandemic and its impact on care complexity in adverse groups.

Wave 2 could have been more negatively impacted than Wave 1 regarding care provision for several reasons. More patients may have been placed on waiting lists due to a lack of capacity or were unsuitable for early rehabilitation treatment. Deconditioning due to the lockdown measures could have also limited service engagement [21, 22]. This may also be linked to increased vulnerability at admission. Regardless, evaluating these patient states without analysing underlying patient frailty markers is difficult. On the other hand, it is also possible that COVID-19 impacted patient function less significantly during Wave 2, and patients did not require extensive rehabilitation and care services. Care variation increased substantially in the older population, which may indicate a shift in prioritisation in health services. Vaccination also likely contributed to the relatively milder presentations in Wave 2 [23, 24]. However, this data was not routinely available for analysis in this study.

We additionally observed some potential issues within the BPMN diagrams, highlighting the limitations of the process model (Figs. 3 and 4). Firstly, the relaxed noise constraint allowed self-loops for many activities, even though these were excluded in the data preprocessing step. While the noise threshold $\theta$ reduces model complexity and limits infrequent behaviour to discover more refined patterns, it can introduce self-loops that deviate from the event log in the current setup. For example, periodic nurse monitoring combined with physiotherapist checkups are common occurrences in practice. Limiting behaviour across each cut operation through the IMi model can indirectly result in self-loops. Wave 2 did not highlight many general aspects of multidisciplinary care typically present during a hospitalisation episode. It was surprising to see many captured events happening independently. Ultimately, we can also attribute some of the inconclusive evidence to the underlying limitations of the extracted EHR data. As patients in Wave 2 were sampled over a longer period, this could partially be attributed to an imbalance in the variation of care sequences between the two cohorts. The range of activities and care providers in the dataset might not be sufficient to derive clinical insights. In hospital services, many other events linked to categories such as laboratory testing, prescribing, bed allocation, and surgery can considerably impact the recovery trajectory.

Process mining continues to be a rapidly evolving field, and its applications on EHR data can significantly aid clinicians and stakeholders in understanding what drives changes in treatment trajectories. Process models, such as IMi, can simplify and deconstruct sequences of care patterns, identifying important interactions between disciplines or lack thereof. We can measure adherence across subgroups, treatment levels, and cohorts through conformance checking and graph similarity measures. In clinical practice, this can pinpoint bottlenecks in complex cases over small sample sizes. PM approaches can also be fine-tuned to consider existing insights from the hospital practice, incorporating them as rules before process discovery.

This case study can serve as a starting point for future research incorporating PM and clinical oversight to generate interpretable clinical pathways across many clinical domains. Operational adherence can be defined in many ways using various quantitative measurements. The appropriate metric is challenging to define, especially in episodes of care with high variability. Therefore, using human judgement might be beneficial, especially where contradictions exist between different quality measures. Designing input rules in the COVID-19 domain is challenging. However, through more well-defined pathways in other long-term conditions (e.g. hip fracture and stroke care), we can look at adherence in subgroups and evaluate COVID-19 status as a confounding variable. Future data studies could expand this set of contact events to incorporate other routine data sources such as ward and speciality movements, blood testing, and comparisons with non-COVID populations. In this study, we focused on hospital survivors, but a statistical approach to estimating competing risks between in-hospital death and resource complexity (e.g. a modified threshold of the GED) could add to understanding across all patients. An updated cohort including events from later waves, vaccination status, and identification of COVID-19 variants could also help provide more robust interpretations of these data.

## Related Work

The use of EHR-based analytics has been prominent in patients with COVID-19. Previous large-scale studies have used routine data to observe disease trajectories, treatment progression, and clinical outcomes [25]. Recent studies have also identified a “long-COVID” population (patients with persisting post-infection symptoms) in Scotland, using linked primary and secondary care data [26]. Previous work has also compared Wave 1 and 2 outcomes in older adults, highlighting lower frailty levels and improved mortality outcomes later in the pandemic [27]. Despite this body of research, patterns of contact with health and care providers are rarely observed due to the small amount of timestamped data recording inpatient activity. Outside of COVID-19, previous studies have collected basic nursing care activities in hospitalised patients [28]. In rehabilitation medicine, studies have retrospectively collected continuous intensity measures, such as the frequency and duration of physiotherapy visits, correlating them to mobility and the likelihood of home discharge [28–31]. Although these measures can serve as proxies for care complexity, it is well-known that most patients with high rehabilitation needs require input from more than one specialist (“multidisciplinary care”), which is not captured in these studies.

In the context of COVID-19 care pathways, PM studies have adapted DFGs in combination with traditional statistical analyses to model disease progression in patients, including events associated with acute rehabilitation care [32]. However, in this instance, there are no strict rules regarding the timing or visit sequences between different care providers, so the use of weighted DFGs would be less meaningful for clinical interpretation. Additionally, filters used to simplify DFGs are known to result in misleading interpretations that deviate from the real output of the process model [33]. Instead, variants of Inductive Miner, which deals with infrequent behaviour in multiple stages of process discovery, have been the preferred method for subpopulation analysis [33–35]. The ability to capture more complex relationships in timestamped data throughout the stages of cut detection, log splitting, and identifying base cases ensures a good balance between model precision and log fitness. Additionally, the IMi algorithm allows for robust filtering of infrequent behaviour across these operations to increase computation speed while reducing model complexity and the risk of overestimating process deviations [36]. HM is another alternative adapted to observe hierarchical patterns and underlying causal dependencies between nodes with comparable performance [37]. Other approaches in handling comparisons between trace executions using alignment-based techniques, [38] rather than token-based replay, have generally been more prominent in practice (resulting in more efficient classification of transitions through “move” events). However, based on our experience, these were computationally challenging to perform over a large population of events with high trace variation and many subgroups.

A similar approach to our subgroup analysis was also applied in a cancer care population by Marazza et al. using an Inductive Miner variant [39]. They used the MIMIC-III dataset to discover variations in treatment pathways across patient subgroups. We provided additional measurements of the coverage rates (estimated as the percentage of fitting traces between groups 1 and 2) to distinguish how well pathways align between complex and less severe cases (Appendix B.4). GED has been previously used in several domains, including information retrieval and machine learning, for pattern recognition to merge similar embeddings or measure the classification accuracy in documents [40, 41]. To our knowledge, this is the first study that provides a comprehensive and validated PM approach on EHR data to compare multidisciplinary care patterns in patients with COVID-19, using multiple levels of granularity.

## Conclusion

Health and care pathways in the hospital are complex for many patients, including those recovering from acute COVID-19 infection. Process mining can aid in providing visual interpretations of treatment progression in these patients and help identify changes in patterns of care. Our approach uses novel routine data from the EHR to gather care provider inputs and linked activities describing the delivered interventions. Using these data, we described the process flows and underlying patterns of care, highlighting some of the distinctions in activity trajectory between Waves 1 and 2 of the pandemic. We applied token-based replay and graph similarity measures to provide an extensive evaluation of the differences in impact on adverse and non-adverse subgroups. This approach can drive further development of novel process-driven applications for understanding treatment policy changes and provide actionable evidence for reducing risks of disruption in healthcare services.

## Supplementary Information

Below is the link to the electronic supplementary material.

Supplementary file1 (DOCX 8262 KB)

## Data Availability

The data that support the findings of this study are not openly available due to reasons of sensitivity, but summary data and visual diagrams can be provided by the corresponding author upon reasonable request. Data and models are located in a Secure Data Environment provided by the DataLoch service.
