# Supplementary material for: Comparing Care Pathways Between COVID-19 Pandemic Waves Using Electronic Health Records: A Process Mining Case Study
Source: J Healthc Inform Res. 2024 Dec 23;9(1):41–66. doi: 10.1007/s41666-024-00181-6 (PMC11782707; doi:10.1007/s41666-024-00181-6)
Supplement: Supplementary file 1 — Supplementary file1 (DOCX 8262 KB) [file 41666_2024_181_MOESM1_ESM.docx]

**APPENDIX**

**Comparing care pathways between COVID-19 pandemic waves using electronic health records: a process mining case study**

Konstantin Georgiev^1^, Jacques D Fleuriot^2^, Petros Papapanagiotou^3^, Joanne McPeake^4^, Susan D Shenkin^5^, Atul Anand^1^

^1^ BHF Centre for Cardiovascular Science, University of Edinburgh, Edinburgh, UK

^2^ Artificial Intelligence and its Applications Institute, School of Informatics, University of Edinburgh, Edinburgh, UK

^3^ Independent Researcher, Edinburgh, UK

^4^ The Healthcare Improvement Studies Institute, Department of Public Health and Primary Care, University of Cambridge, Cambridge, UK

^5^ Ageing and Care Research Group and Advanced Care Research Centre, Usher Institute, University of Edinburgh, Edinburgh, UK

**Short Title:** Process Mining COVID-19 Care Patterns

**Corresponding author:**

Konstantin Georgiev

University of Edinburgh/BHF Centre for Cardiovascular Science

Chancellor’s Building

49 Little France Crescent

Edinburgh EH16 4SA

United Kingdom

Email: [K.S.Georgiev@sms.ed.ac.uk](https://d.docs.live.net/b2b73a5e8ffa31df/Documents/UoE/COVID-19%20rehab/process_mining/rebuttal/K.S.Georgiev@sms.ed.ac.uk)

# Appendix A. Supplementary Methods

## Appendix A.1. Process Discovery techniques

**Alpha Miner**

Alpha Miner is one of the simplest and earliest-developed PM algorithms that can discover concurrent transitions using Petri Nets.^1^ However, its lack of precision and limitations in capturing only direct relations in the event log makes it less useful in practice but still beneficial for baseline comparisons. It presents a very simple approach which only manages to capture directly-follows relations in a Petri Net.^1^ For each pair of nodes $(N_{i}, N_{j})$ in the event log $L$, if there is a direct succession between $N_{i}$ and $N_{j}$, a transition $y$ and two places $x_{i}$ and $x_{j}$ will be appended to the Petri Net $P$, where the flow relations will also be updated to include $(x_{i}, y)$ and $(y, x_{j})$. If $N_{i}$ has no predecessors, $y$ will only be added to the flow relation of $P$ along with $x_{i}$ and the flow relation will be only $(y, p_{i})$. Finally, if $N_{i}$ has no successors, only $(x_{i}, y)$ will be appended to the flow relation, along with the place and transition.

**Inductive Miner**

Inductive Miner is a significantly improved model that detects cuts and recursively identifies base cases that apply to individual partitions of the event log.^2^ This results in the ability to capture more complex relationships between activities, described as hierarchical dependencies, as well as potential fall-throughs in the process flow. The Inductive Miner infrequent (IMi) provides a way of locally dealing with infrequent behaviour across each cut operation and log split, significantly improving precision and the speed of discovering a Petri Net.^3^ Let $f(N_{i}, N_{j})$ denote the frequency of a directly-follows relation, expressed as $N_{i}\to N_{j}$. The Inductive Directly-follows Graph (IDFG) Miner operates by identifying cut sets directly from the DFG, separating it into partitions.^4^ It discovers rules by recursively splitting a directly-follows graph of the pathway in partitions until a set of base cases is defined (containing only a single event after completing execution).^4^ Similarly to IMi, it has built-in techniques to handle infrequent behaviour, which may significantly impact the discovered rules. The algorithms will then combine these sets into a process tree, or a hierarchical representation of the flow relations between the cut sets. Finally, this will be converted into a Petri Net 𝑃 in a notation describing places and transitions. The threshold $\theta_{IMi}\in[0,1]$ (lower value indicates a stronger adjustment) is used to locally eliminate infrequent traces across all cut operations. Meanwhile, the threshold parameter $\theta_{IDFG}$ is used to eliminate edges ($E_{i,} E_{j}$), where their relative frequency $f\left( E_{i}, E_{j} \right)<\theta_{IDFG}\times\max_{k} (\left| (i,k) \right|)$, where $E_{k}$ is the most frequent outgoing edge with a direct relation to $E_{i}$.

**Heuristics Miner**

Heuristics Miner is another PM algorithm that utilises a DFG but is less prone to noise due to the use of dependency graphs to model relationships between nodes.^5^ Unlike Inductive Miner, it only records the sequence of events rather than the frequency, through causal matrices, which can identify ‘hidden’ long-distance relations. It detects less restrictive dependency relations, which indicates that $N_{i}\Rightarrow N_{j}$, that is $N_{i}$ often follows $N_{j}$. The degree of significance of this relation can be treated as a threshold parameter $\theta_{HM}\in\left[ 0,1 \right]$, where similarly to limiting infrequent behaviour in IMi, this is used to prune weak dependencies in the model. The next step converts the DFG into a dependency graph, displaying the nodes $N$ and their causal relationships. Additional metrics can be applied, such as dealing with length-two loops to improve precision in long-distance relations.

## Appendix A.2. Conformance Checking and Graph Similarity

We use a modified version of the token-based replay algorithm to measure conformance of a discovered Petri Net against its original event log, as well as cross-log conformance between two cohorts.^6^ As per Petri Net rules, a token is an entity that accumulates within places ($x_{1}, x_{2}, \ldots, x_{n}$) and is consumed or produced, only when a connected transition $y$ is enabled (all places preceeding contain at least one token). A trace $t$ of an event log $L$ can be treated as ‘fitting’, if during model execution, any transition $y$ can be fired without the need to insert additional tokens, starting from the initial marking $m_{o}$. Therefore, the final state of $t$ must not have any missing (tokens consumed while $y$ is not enabled) or remaining (tokens produced but never consumed until end of execution) tokens generated from $L$.

To estimate a measure of the overall log fitness of a Petri Net $P$ on an event log $L$, let $t_{i}$ represent the i-th trace in $L$ and $\tau=(t_{1},t_{2}, \ldots, t_{k})$be the execution state for the set of transitions in $t_{i}$. We can then treat the overall number of remaining tokens in the trace as $r(\tau)$, missing tokens as $m(\tau)$, produced tokens as $p(\tau)$ and consumed tokens as $c(\tau)$. The fitness for $t_{i}$ can then be expressed as:

|  | $LF_{i}\left( t_{i},\tau\right)=\frac{1}{2}\left( 1-\frac{m\left( \tau\right)}{c\left( \tau\right)} \right)+\frac{1}{2}\left( 1- \frac{r\left( \tau\right)}{p\left( \tau\right)} \right)$ | (1) |
| --- | --- | --- |

Then, we can estimate this proportion on the log-level by summing over all token instances and obtaining the log fitness:

|  | $LF_{L}=\frac{1}{2}\left( 1-\frac{\sum_{L_{i}\in L} m\left( \tau\right)}{\sum_{L_{i}\in L} c\left( \tau\right)} \right)+\frac{1}{2}\left( 1- \frac{\sum_{L_{i}\in L} r\left( \tau\right)}{\sum_{L_{i}\in L} p\left( \tau\right)} \right), LF_{L}\in[0,1]$ | (2) |
| --- | --- | --- |

Precision is another conformance checking measure which uses prefixes (target node combinations) to estimate how well the process model represents the transitions that follow the prefix.^7^ Precision can be estimated by calculating the proportion of ‘escaping edges’ $|E_{t}\left( \tau\right)|$ not linked to event log behaviour versus the accepted tasks that are captured $|A_{t}\left( \tau\right)|$ for a set of transitions $\tau_{i}$. On the log-level this can be summarised as:

|  | ${PR}_{L}=1-\frac{\sum_{i=1}^{L_{i}\in L} \left\vert E_{t}\left( \tau\right) \right\vert}{\sum_{i=1}^{L_{i}\in L} \left\vert A_{t}\left( \tau\right) \right\vert}, {PR}_{L}\in[0,1]$ | (3) |
| --- | --- | --- |

On the other hand, generalisation validates whether the Petri Net is overfitting to the behaviour described in the event log, based on how frequently transitions in the net are fired. Let $X_{\tau}$ be the overall number of executions for a node $N_{i}$ in $L$. On the log-level, the generalisation can then be expressed as:

|  | $G_{L}=1-\frac{\sum_{i=1}^{L_{i}\in L} \left( \sqrt{X_{\tau}} \right)^{-1}}{\sum_{i=1}^{L_{i}\in L} X_{\tau}}, G_{L}\in[0,1]$ | (4) |
| --- | --- | --- |

We use the Graph Edit Distance (GED) at the subgroup analysis stage as a measure for graph dissimilarity between Petri Nets $P_{i}$ and $P_{j}$. This is expressed as the minimum number of edit operations required to transform $P_{i}$ into $P_{j}$. Using this notation, we represent the Petri Nets as bipartite directed graphs, which allow us to estimate the GED as follows:

|  | $GED\left( P_{i},P_{j} \right)=\min_{q\in p\left( P_{i}, P_{j} \right)} (\sum_{m=1}^{k} c(q_{m})), GED\left( P_{i}, P_{j} \right)\in\left[ 1,\infty\right),$ | (5) |
| --- | --- | --- |

where $q$ is the set of possible graph edit operations (edge insertion, deletion, substitution or node insertion, deletion and/or substitution), $k$ is the list of traversable paths across both $W_{i}$ and $W_{j}$ and $c(q_{m})$ is the cost of applying the current edit operation. By default, each edit operation is assigned a cost of 1, which is accumulated upon traversing the paths.

# Appendix B. Supplementary Results

## Appendix B.1. Data and event log distributions


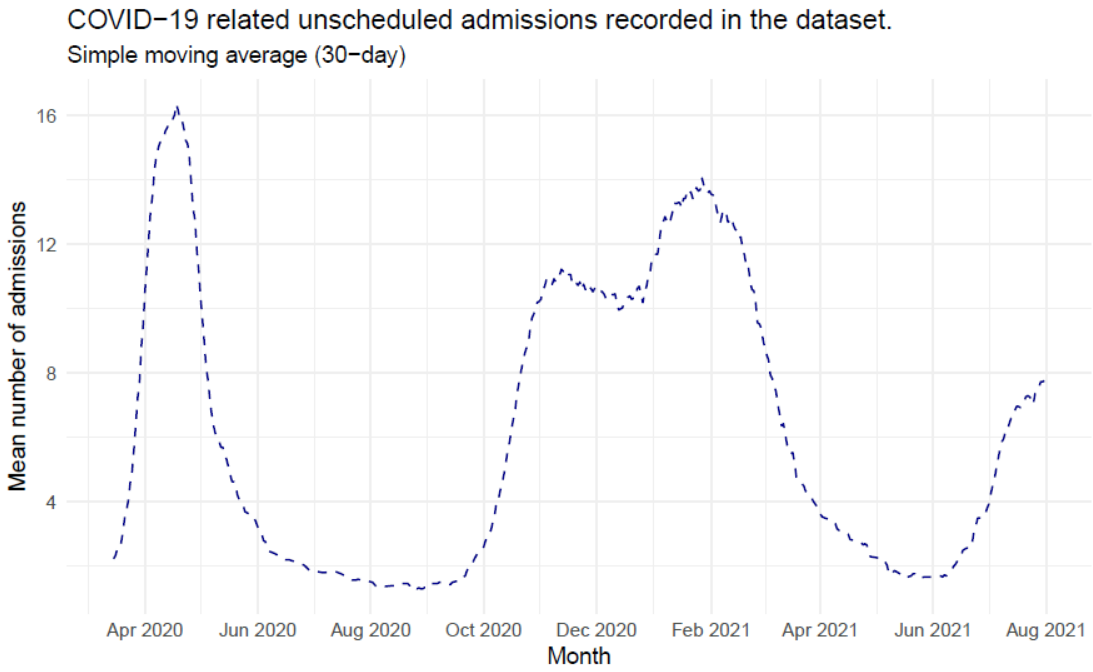


**Figure S1.** Time-series plot representing the recorded peaks in unscheduled admissions within NHS Lothian throughout the first and second COVID-19 waves.


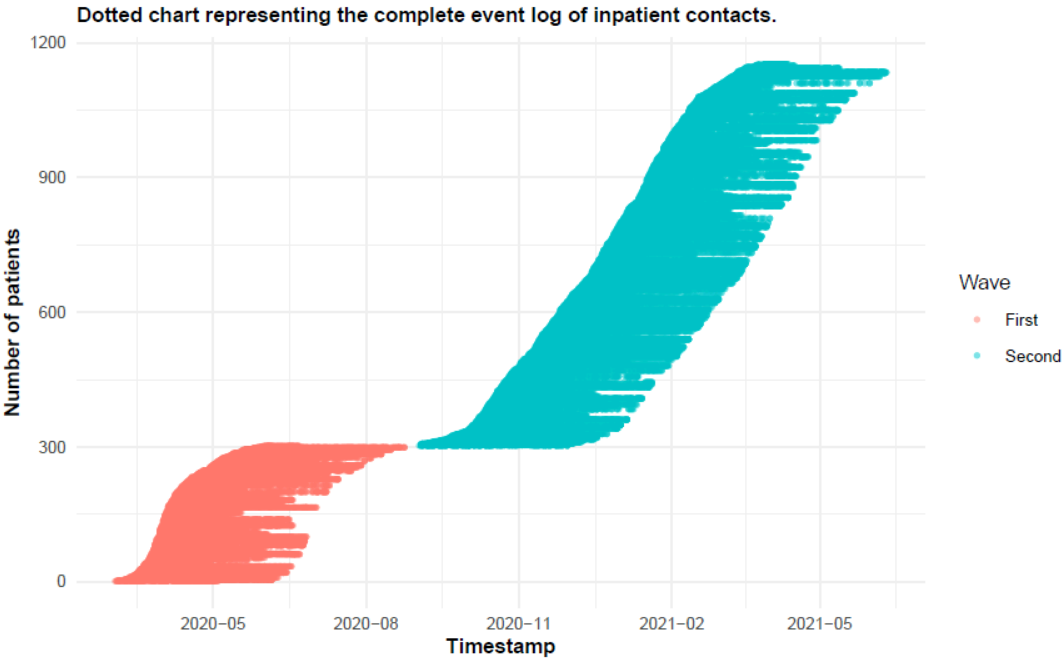


**Figure S2.** Dotted chart plot describing the recorded time of each case on the provider-level.

## Appendix B.2. Generated Directly-follows Graphs (DFGs) used to fit the IDFG Miner

**
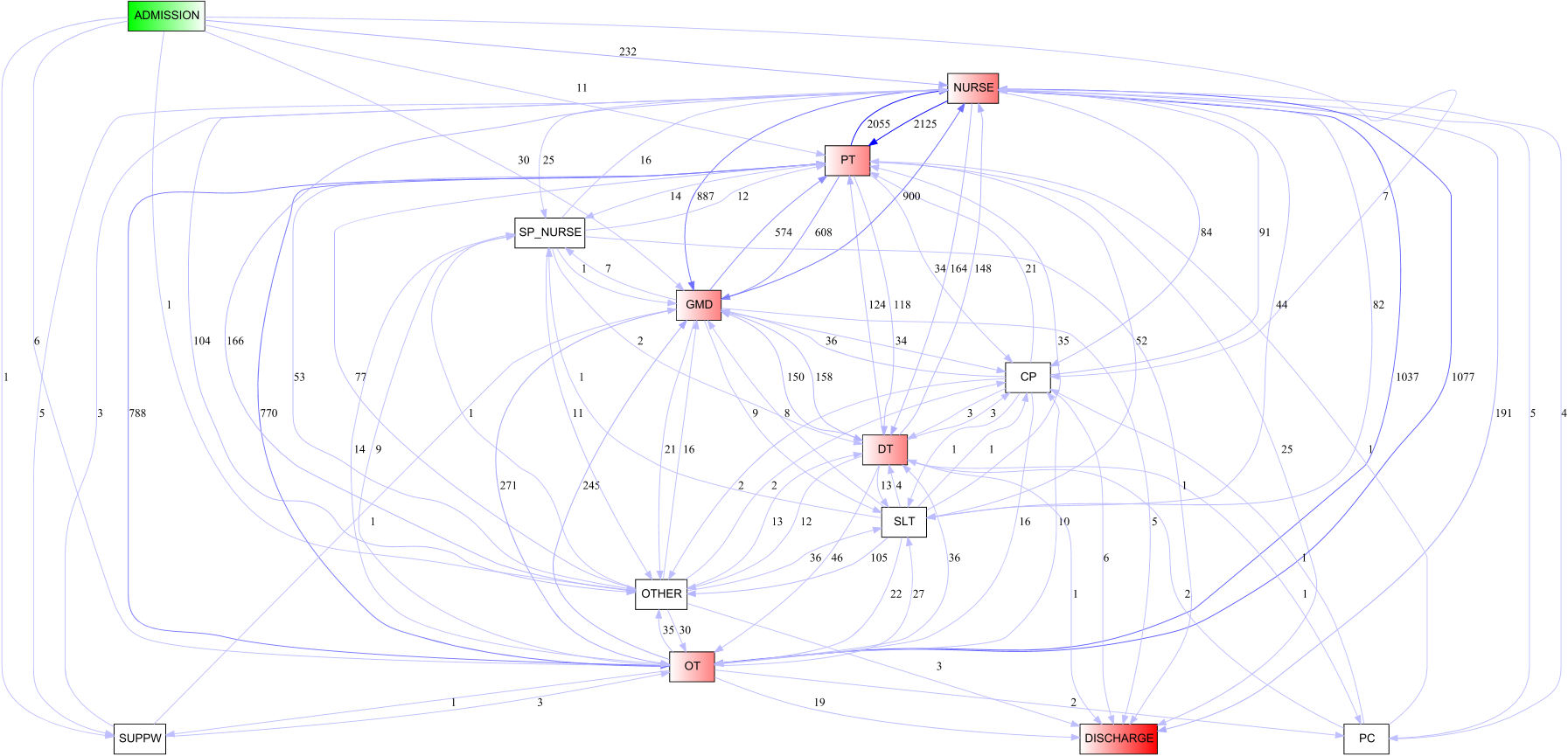
**

**Figure S3.** Complete Directly-follows Graph (DFG) representing the as-is state of the Wave 1 provider-level event log.

**
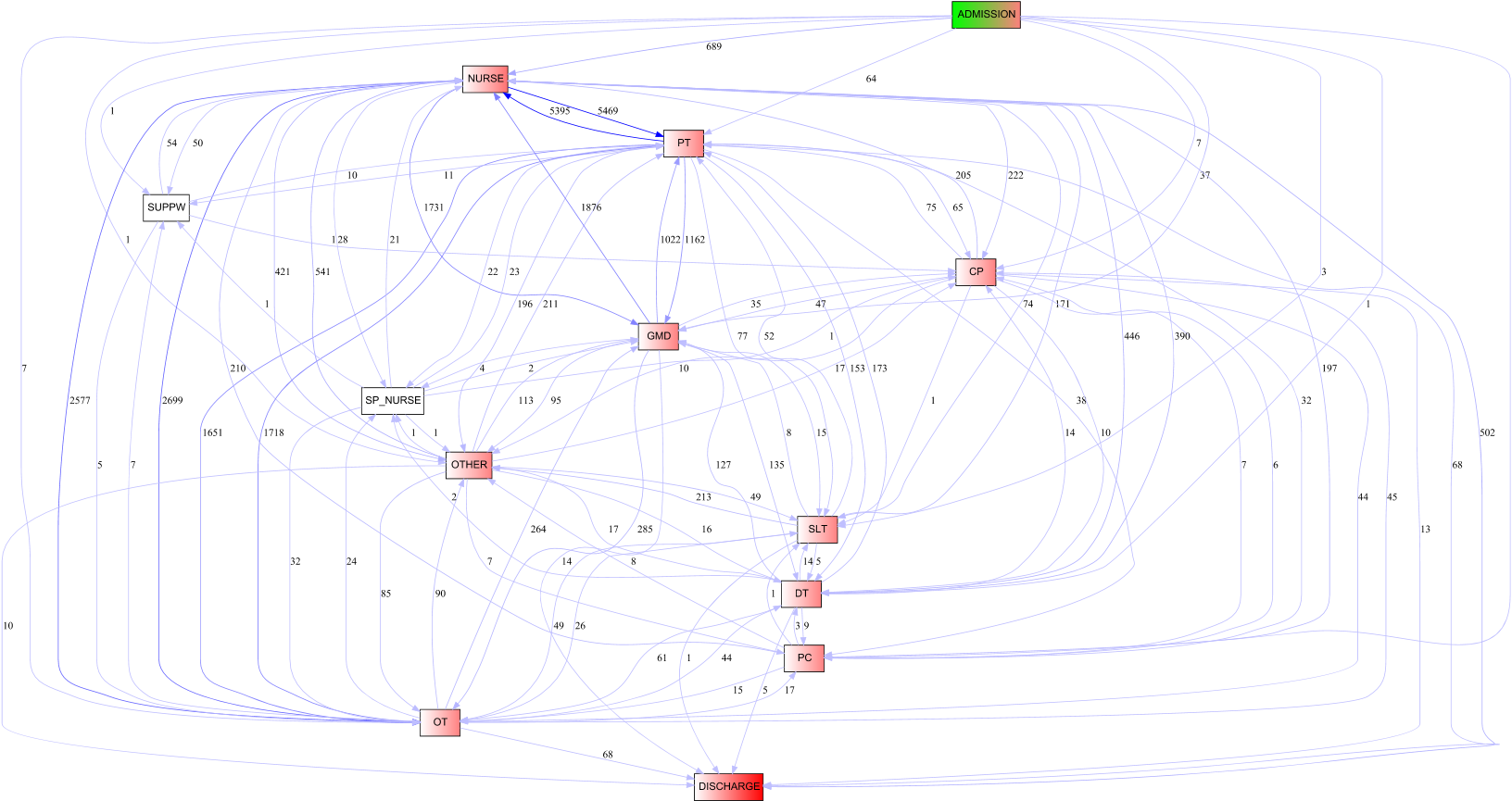
**

**Figure S4.** Complete Directly-follows Graph (DFG) representing the as-is state of the Wave 2 provider-level event log.


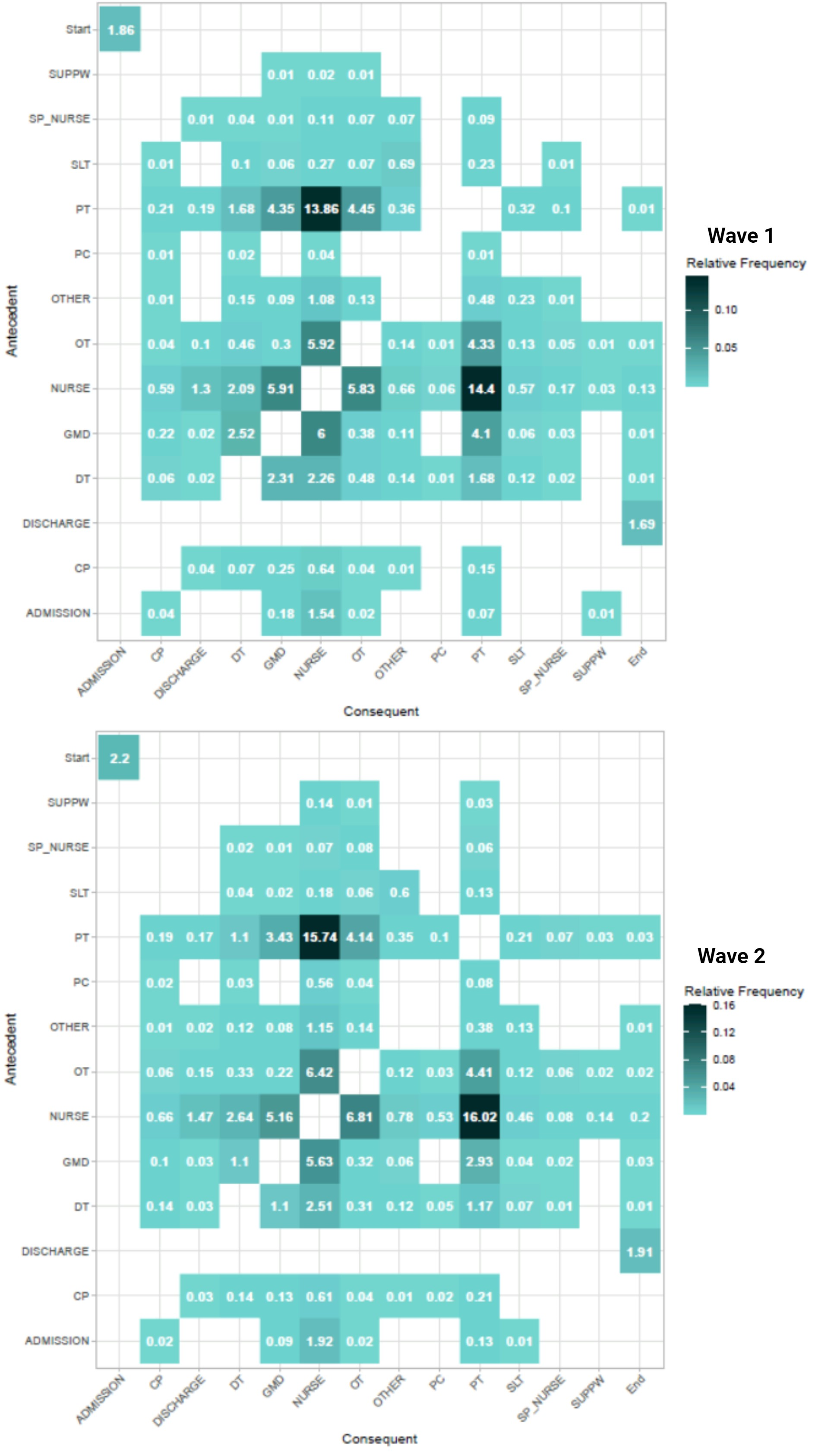


**Figure S5.** Precedence matrices on the DFGs describing the provider-level interactions per wave.


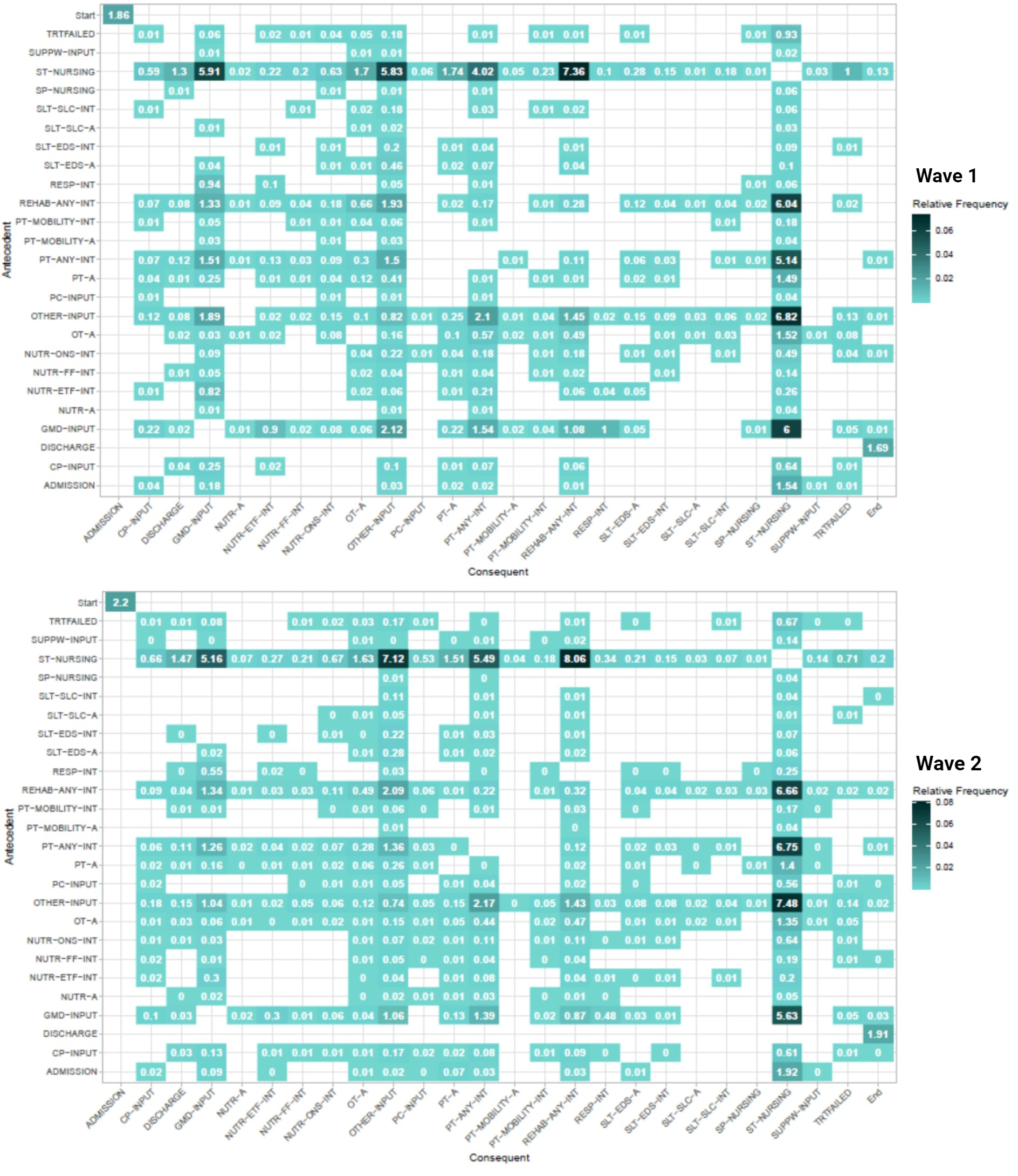


**Figure S6.** Precedence matrices on the DFGs describing the activity-level interactions per wave.

## Appendix B.3. Additional BPMN diagrams representing subgroup contact patterns


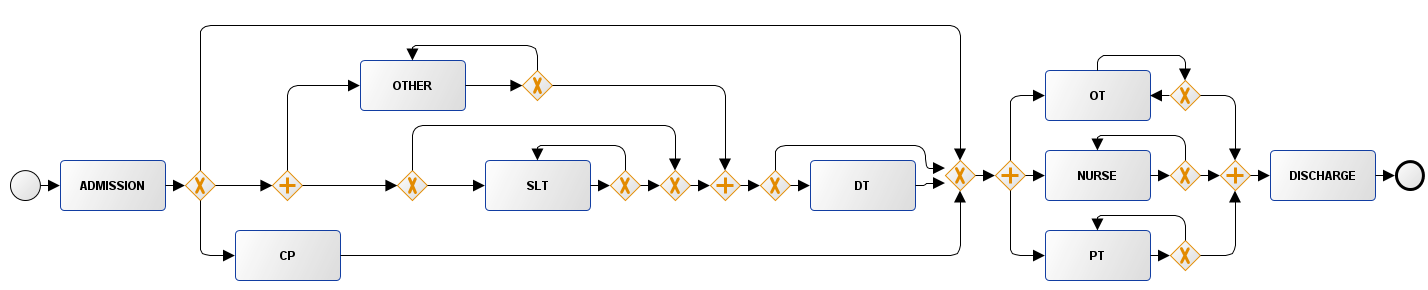


1.
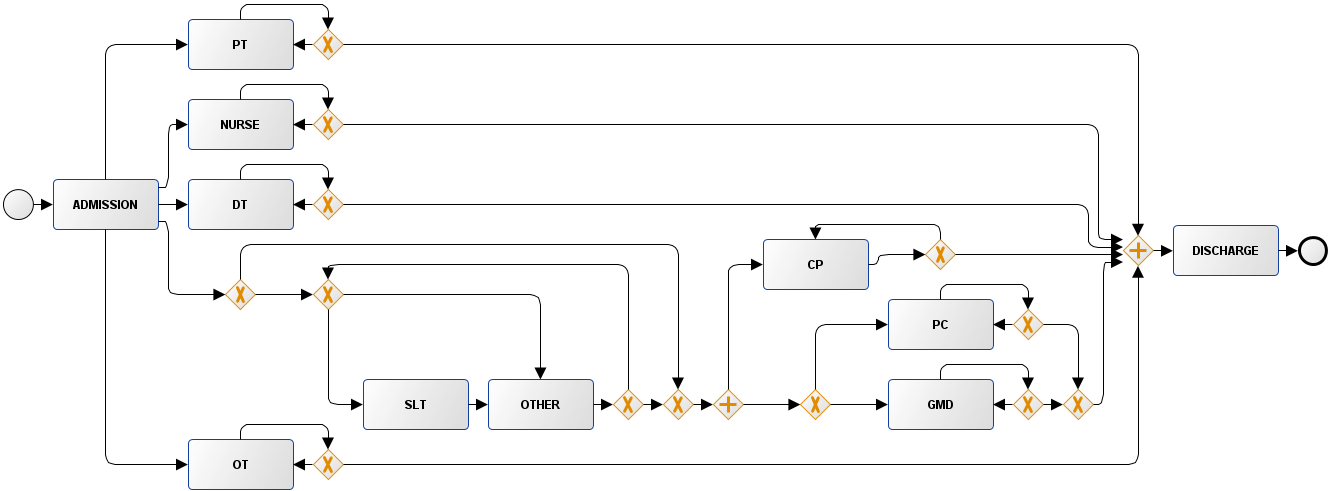
**>75 years subset**
2. **1-year all-cause mortality**


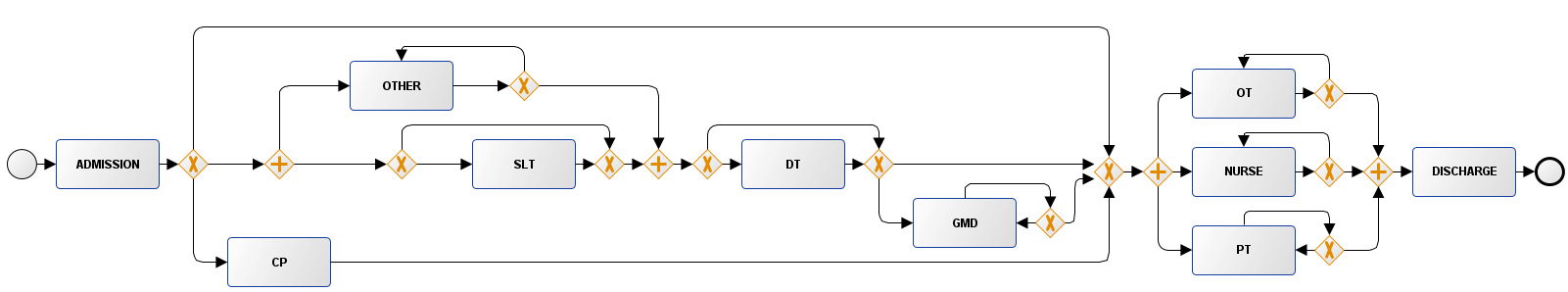
**(c) Multimorbidity**

**
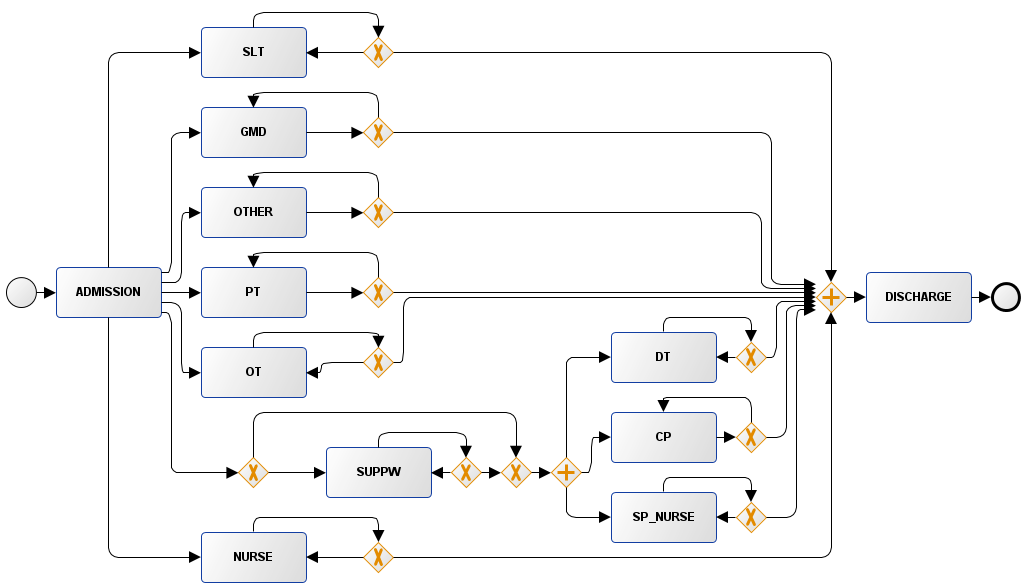
**

**(d) Extended stay**

**
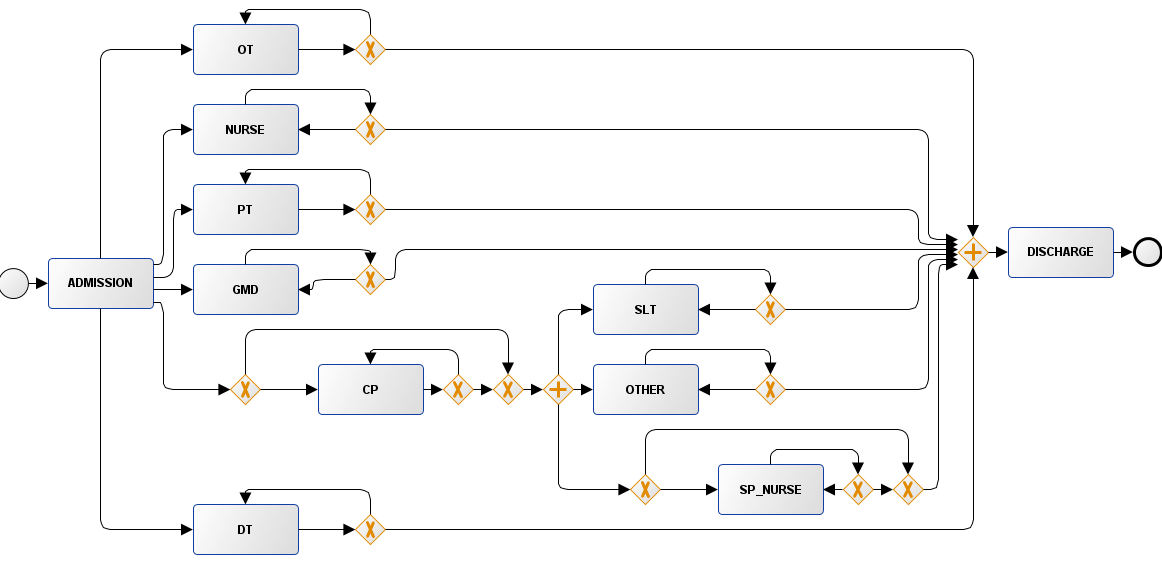
**

**(e) Intensive Therapy**

**
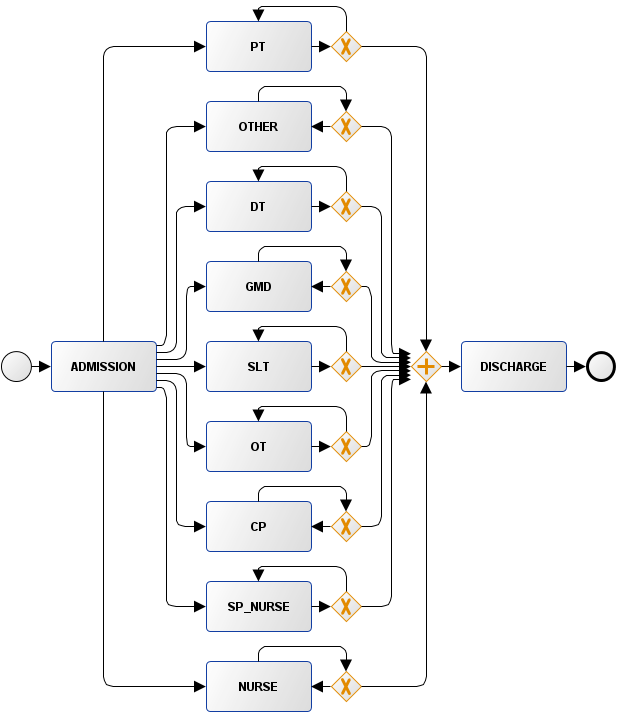
**

**(f) High Out-of-hours care**

**Figure S7.** Provider-level BPMN diagrams discovered with Inductive Miner on the Wave 1 population.


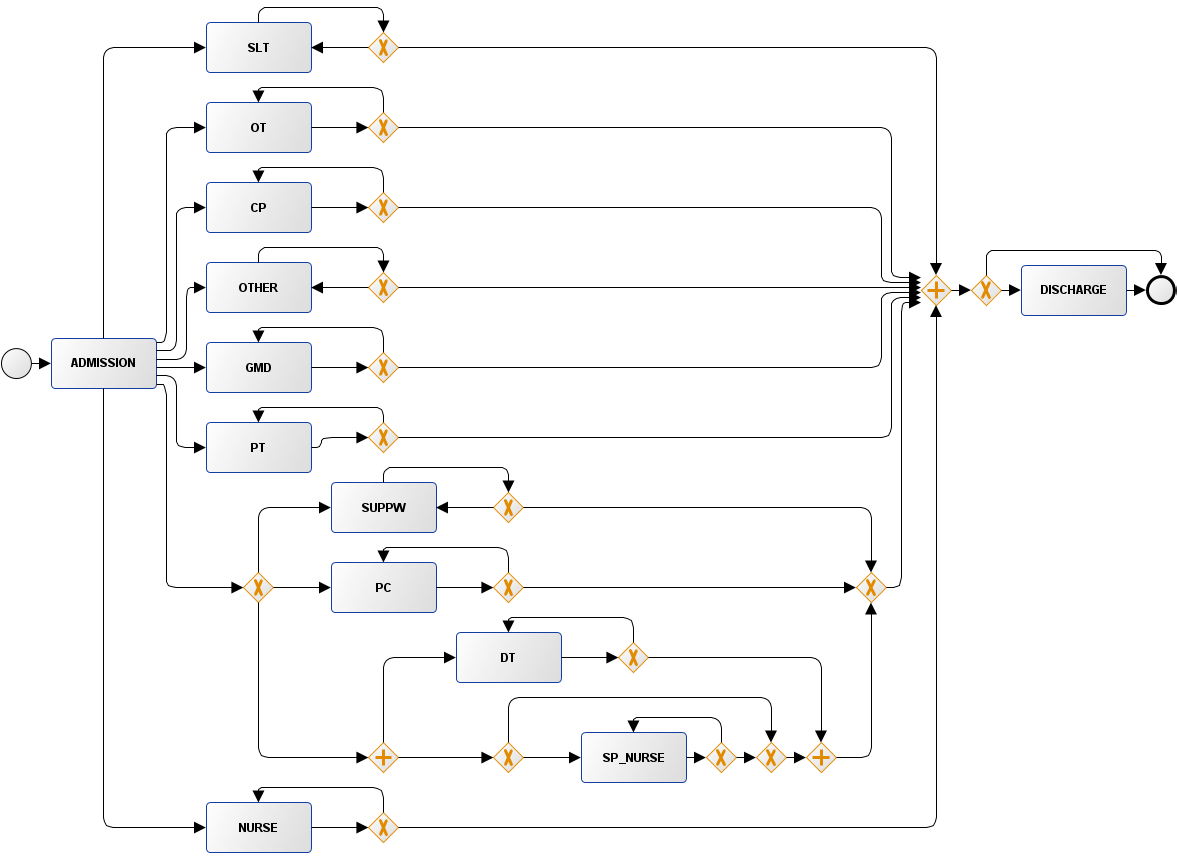

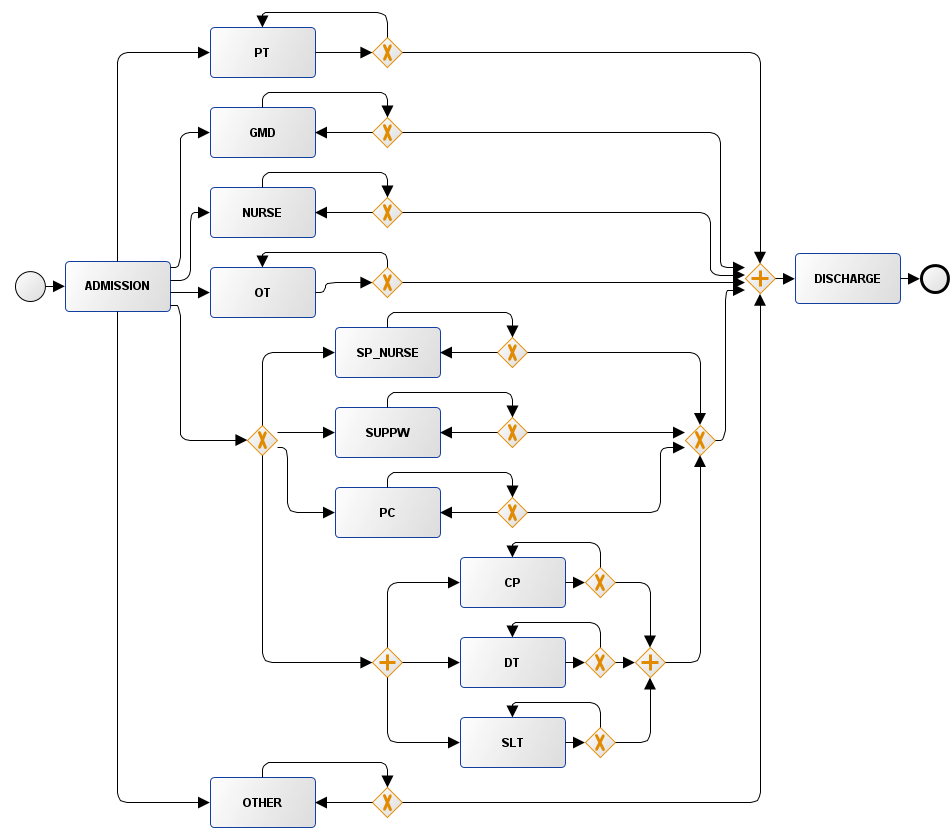


1. **>75 years subset (b) Extended stay**

**
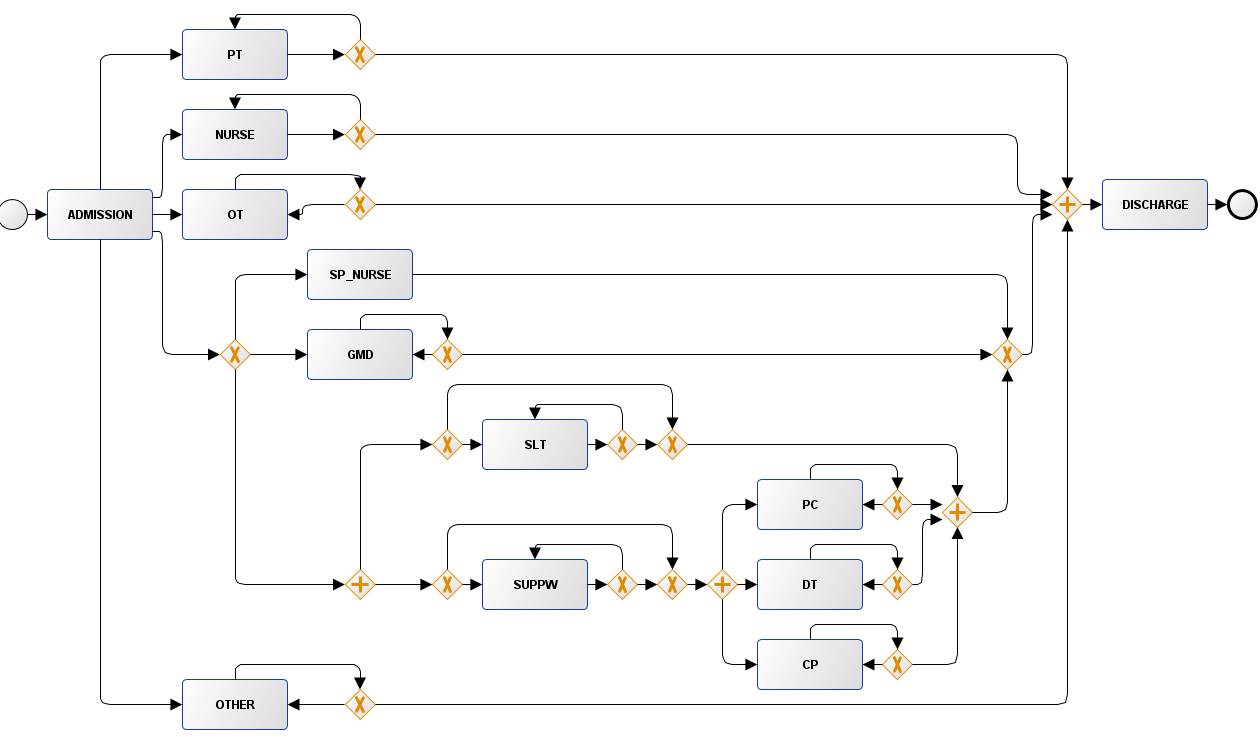
**

**(c) 1-year all-cause mortality**

**
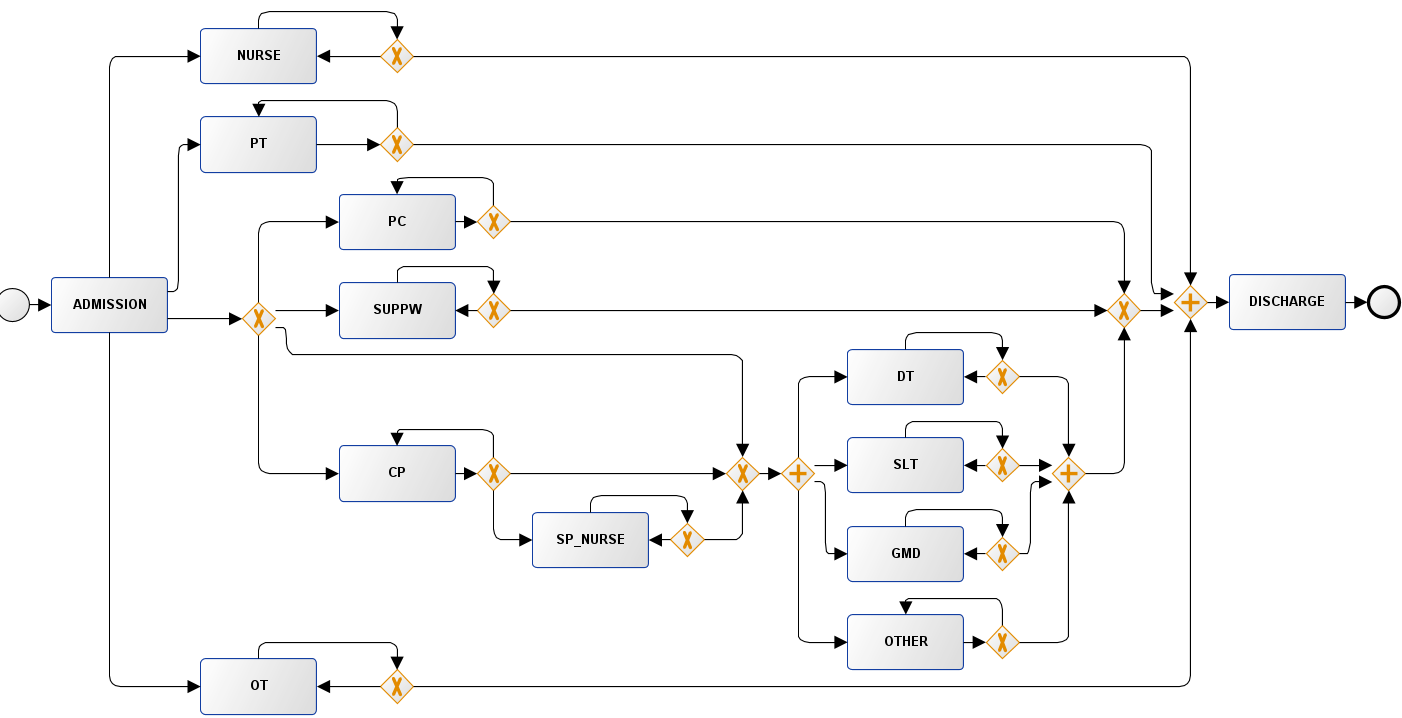
**

**(d) Multimorbidity**

**
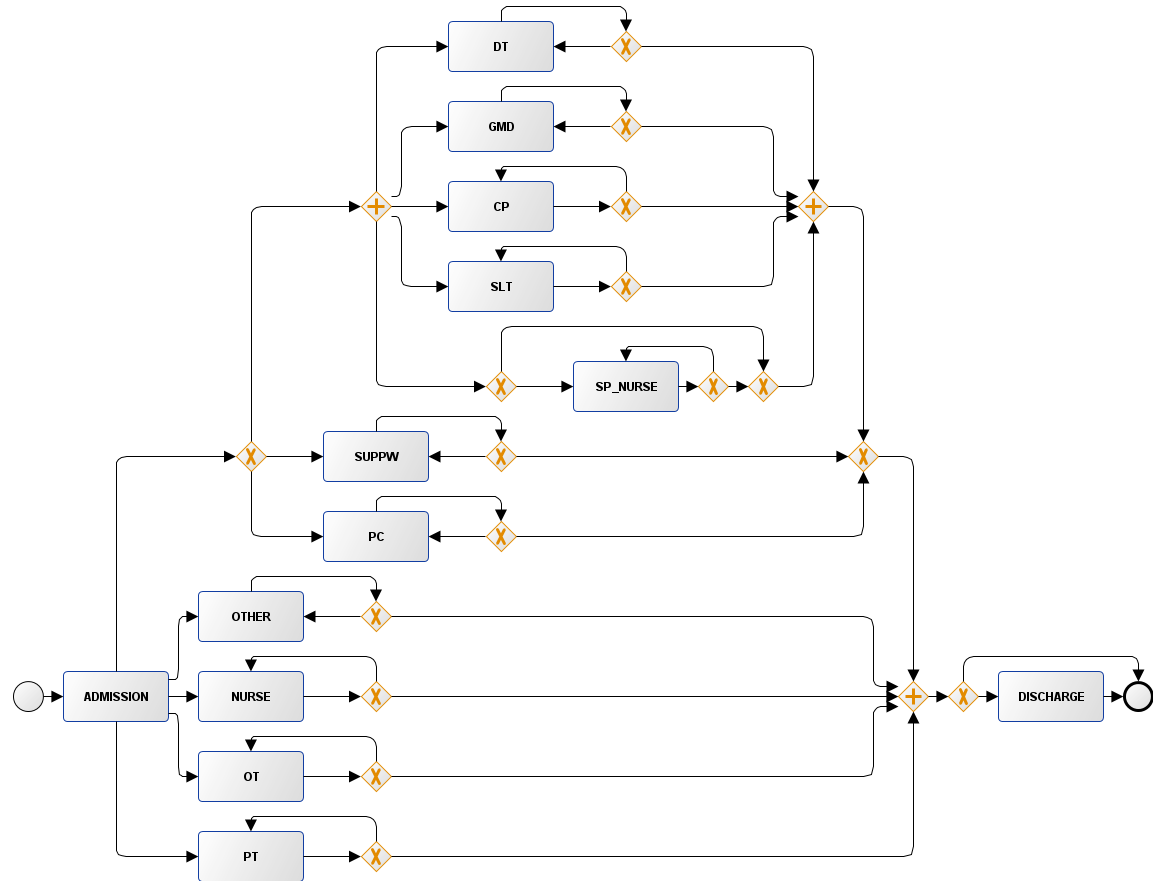
**

**
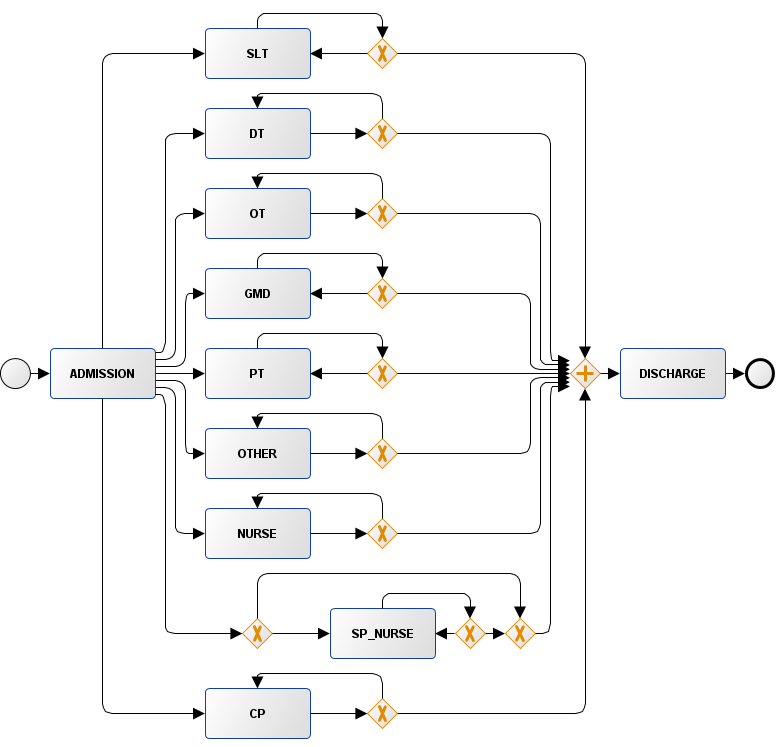
**

**(e) Intensive Therapy (f) High Out-of-hours care**

**Figure S8.** Provider-level BPMN diagrams discovered with Inductive Miner on the Wave 2 population.

**
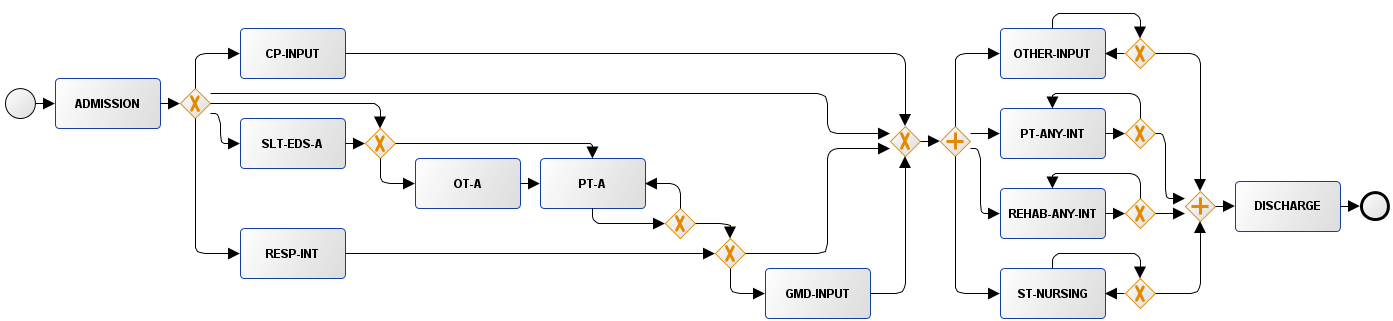
**

1. **>75 years subset**

**
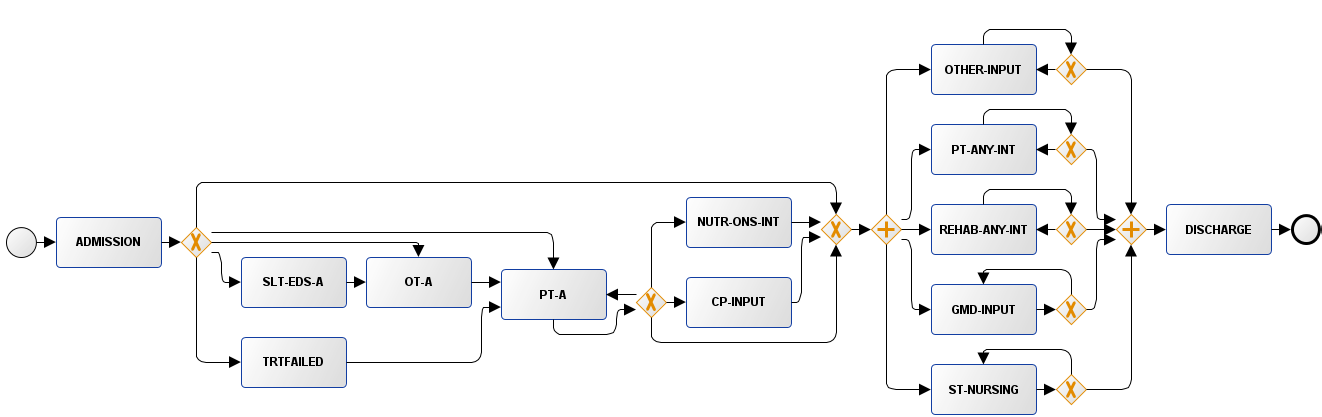
**

1. **Extended stay**


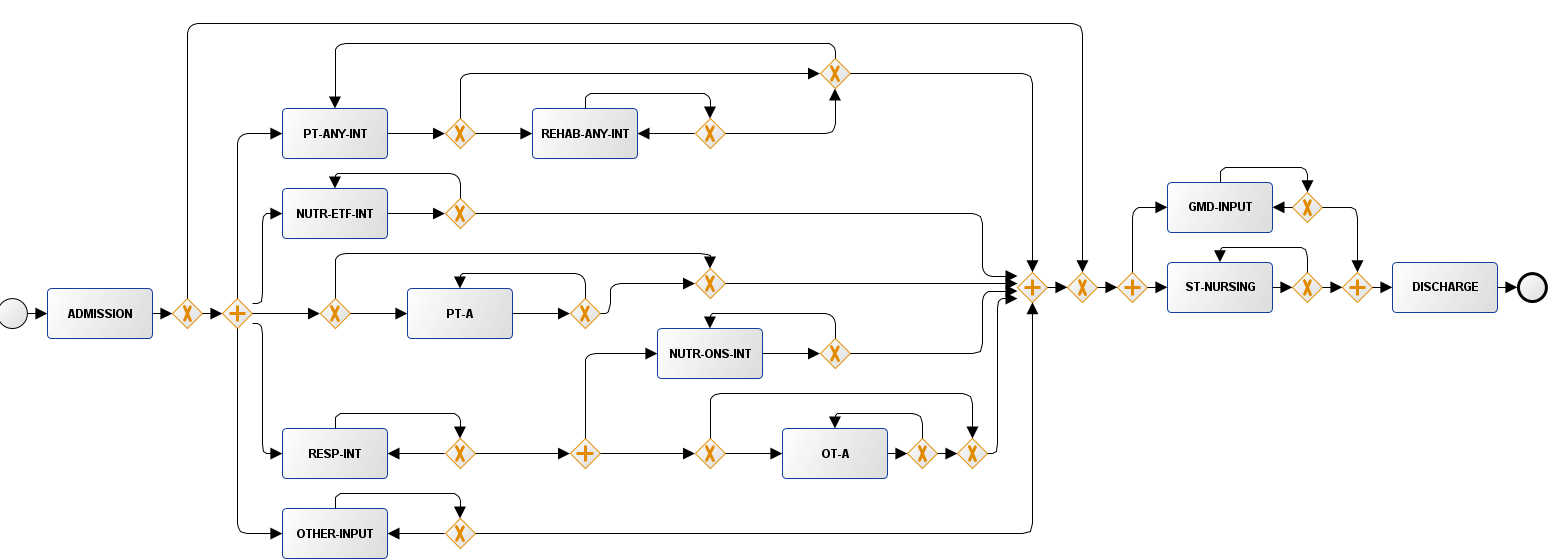


1. **Intensive Therapy**

**
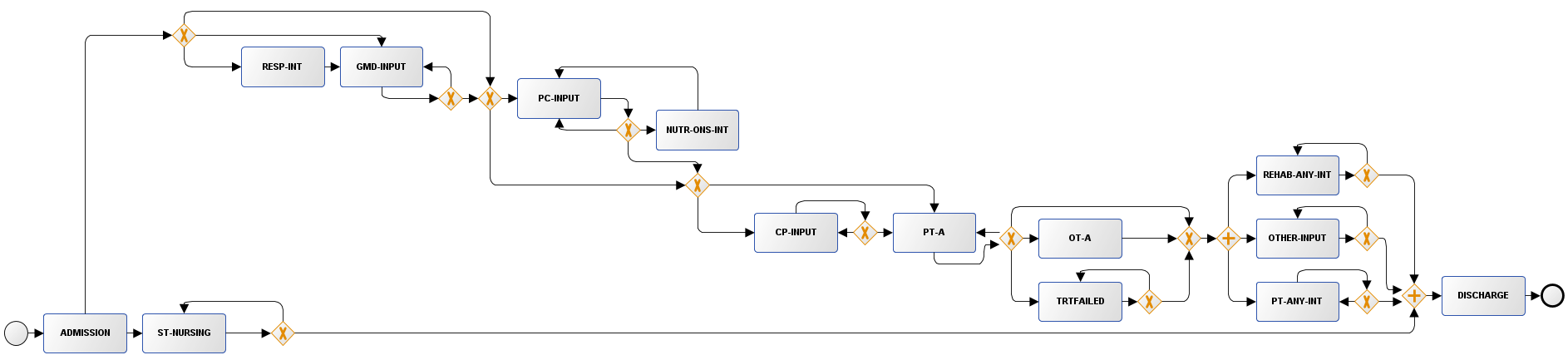
**

**(d) 1-year all-cause mortality**

**
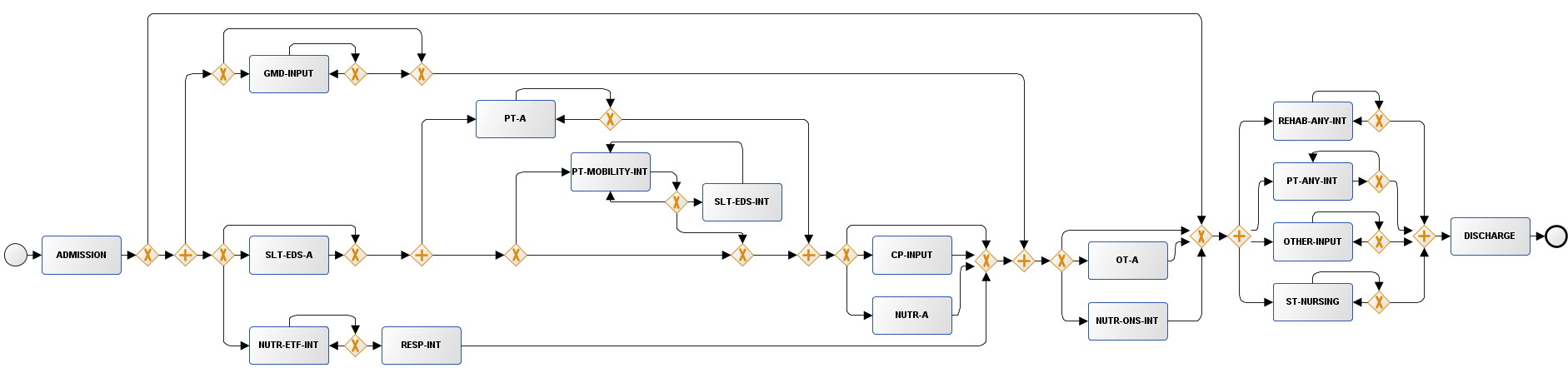
**

**(e) Multimorbidity**

**
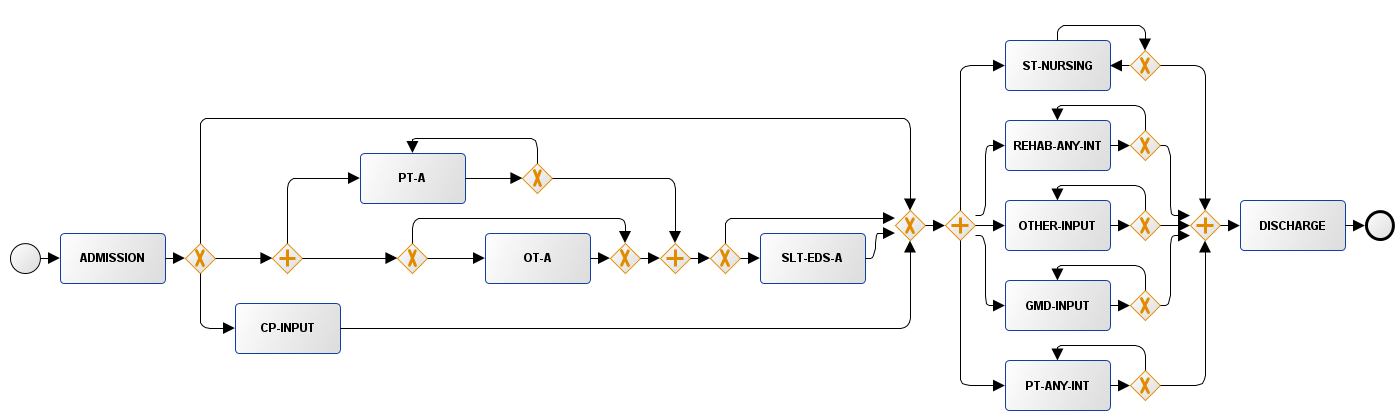
**

**(f) High Out-of-hours care**

**Figure S9.** Activity-level BPMN diagrams discovered with Inductive Miner on the Wave 1 population.

**
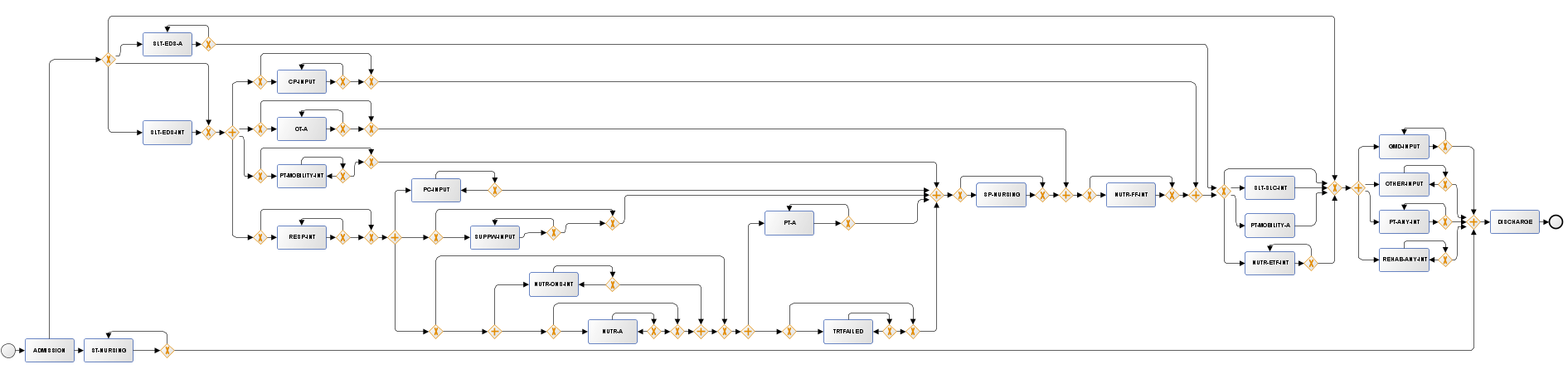
**

**(a) >75 years subset**

**
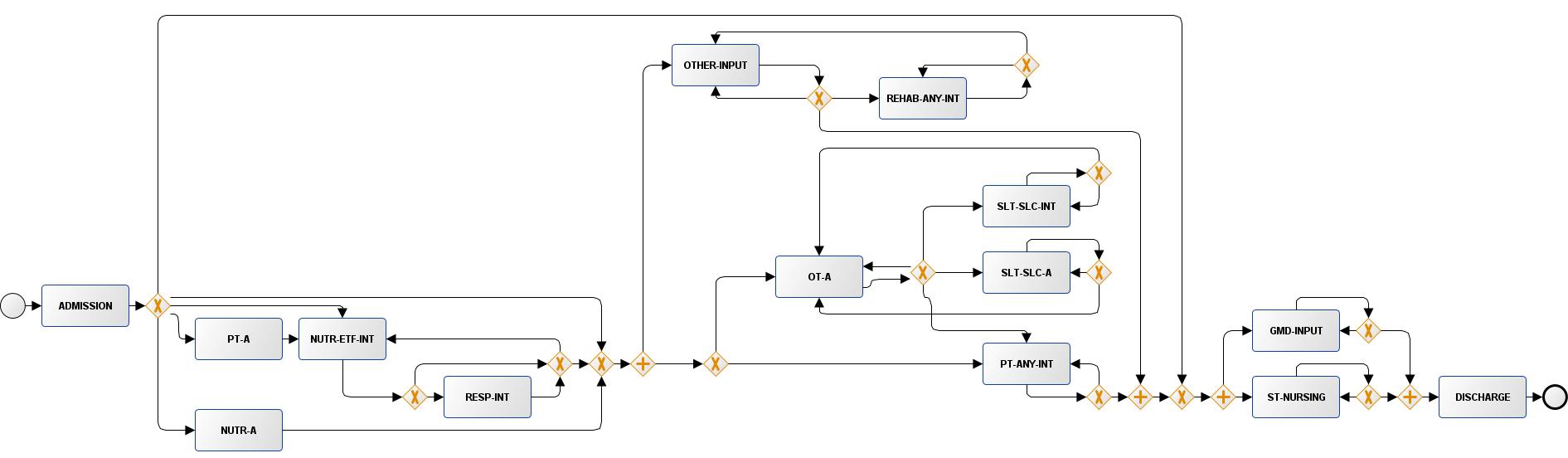
**

**(b) Intensive Therapy**

**
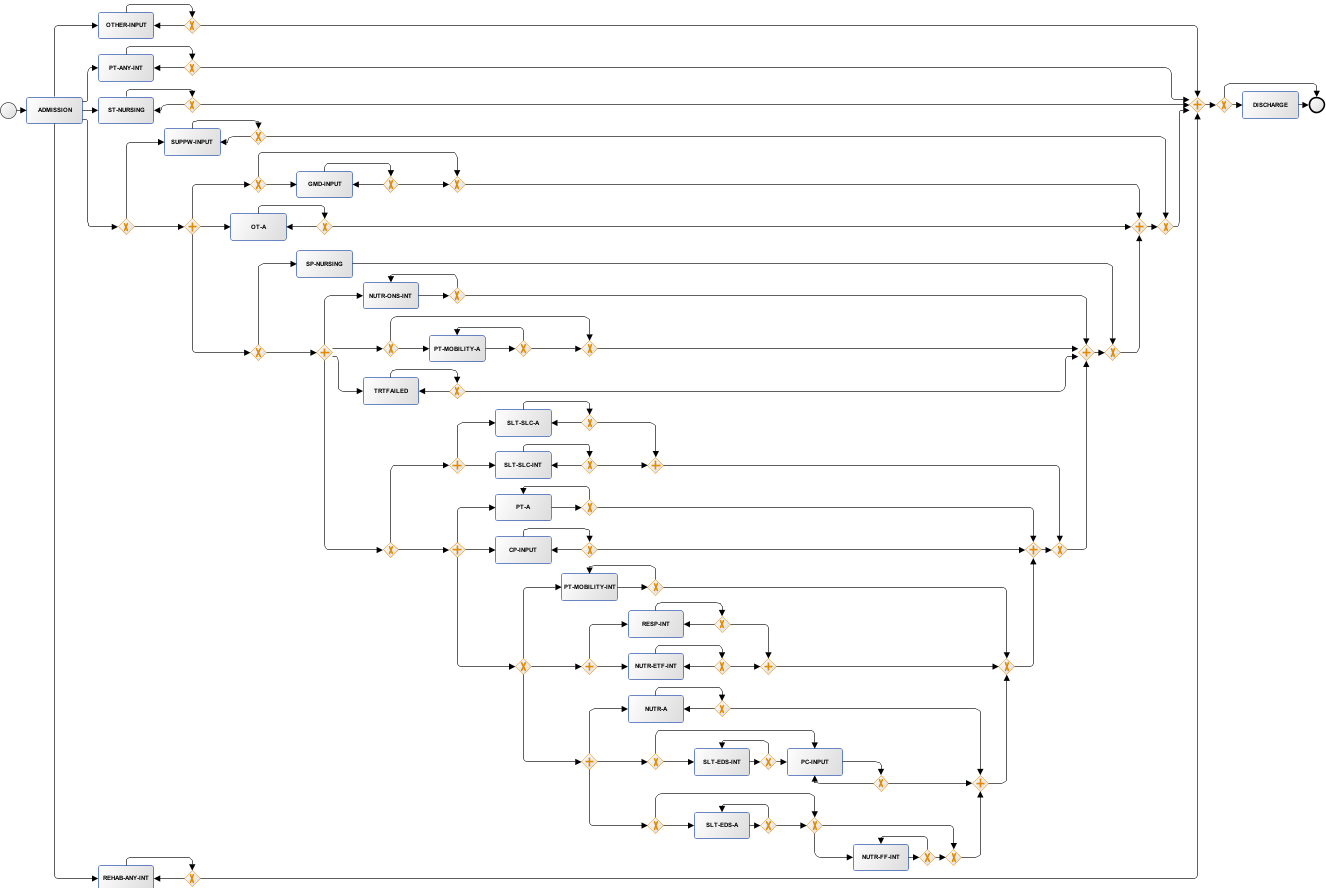
**

**(c) Extended stay**

**
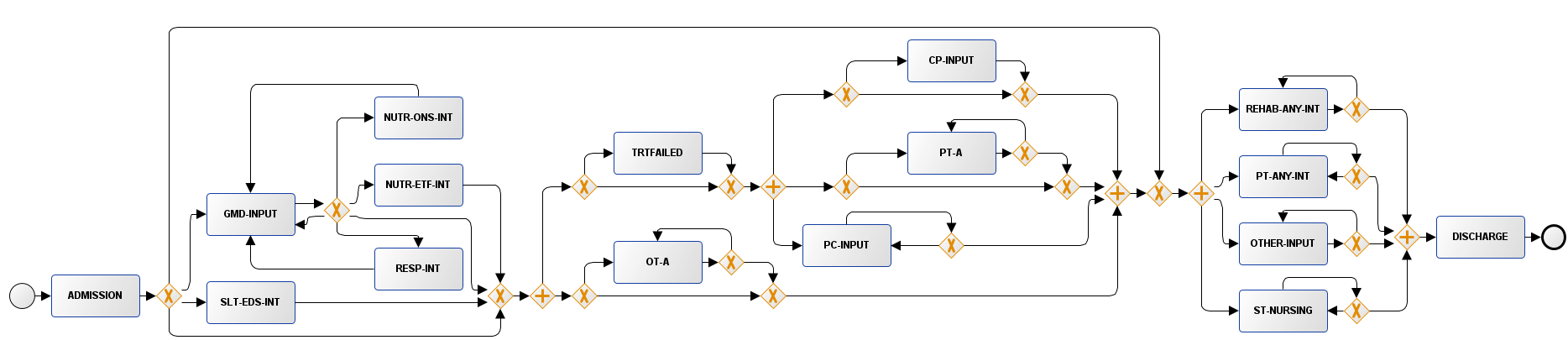
**

**(d) 1-year all-cause mortality**

**
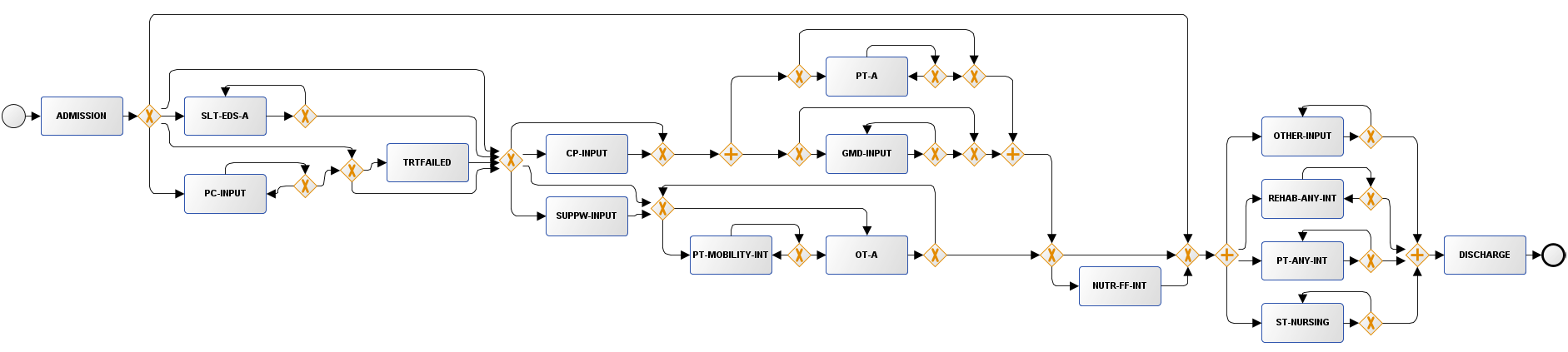
**

**(e) Multimorbidity**

**
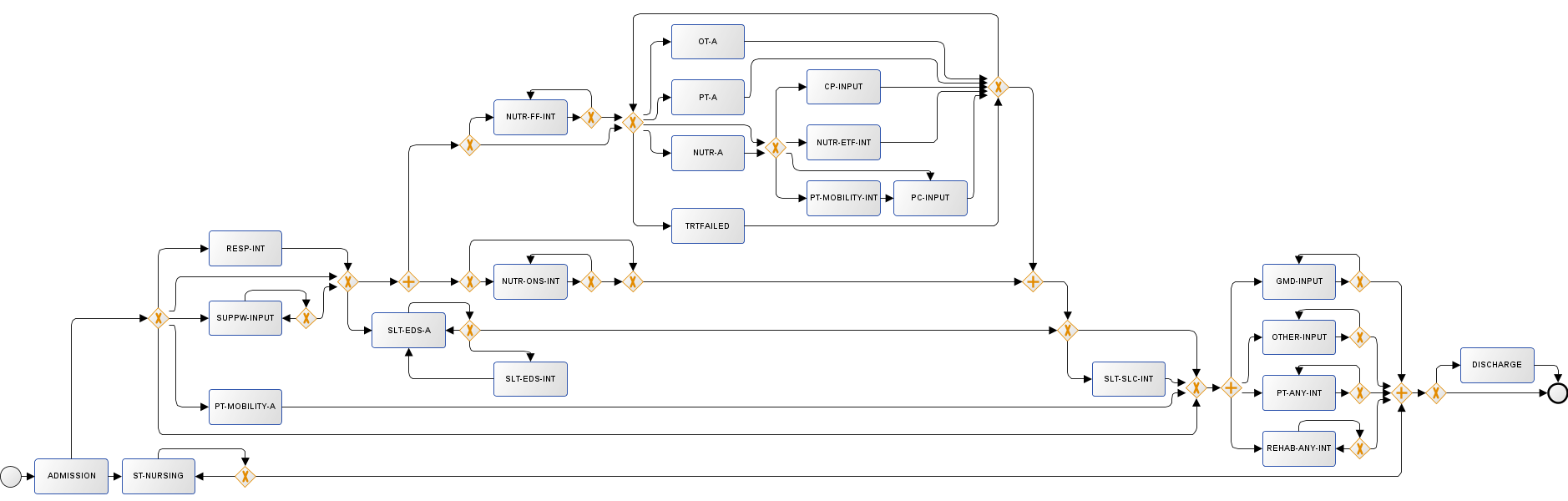
**

**(f) High Out-of-hours care**

**Figure S10.** Activity-level BPMN diagrams discovered with Inductive Miner on the Wave 2 population.

## Appendix B.4. Stratified cross-log conformance checking across sex and socioeconomic status

| Subset | | | | | Log 1 to Wave 2 model replay | | | Log 2 to Wave 1 model replay | | | Mean | | | $\mathbf{GED}$ |
| --- | --- | --- | --- | --- | --- | --- | --- | --- | --- | --- | --- | --- | --- | --- |
|  |  |  |  |  | $\mathbf{LF}\left( {\mathbf{L}_{\mathbf{1}}\mathbf{, P}}_{\mathbf{2}} \right)$ | $\mathbf{PR}\left( {\mathbf{L}_{\mathbf{1}}\mathbf{, P}}_{\mathbf{2}} \right)$ | $\mathbf{G}\left( {\mathbf{L}_{\mathbf{1}}\mathbf{, P}}_{\mathbf{2}} \right)$ | $\mathbf{LF}\left( {\mathbf{L}_{\mathbf{2}}\mathbf{, P}}_{\mathbf{1}} \right)$ | $\mathbf{PR}\left( {\mathbf{L}_{\mathbf{2}}\mathbf{, P}}_{\mathbf{1}} \right)$ | $\mathbf{G}\left( {\mathbf{L}_{\mathbf{2}}\mathbf{, P}}_{\mathbf{1}} \right)$ | $\bar{\mathbf{LF}}$ | $\bar{\mathbf{P}\boldsymbol{R}}$ | $\bar{\mathbf{G}}$ |  |
| Provider-level | **Sex** | **Female** | | **Y** | 0.953 | 0.146 | 0.724 | 0.965 | 0.471 | 0.833 | 0.959 | 0.308 | 0.778 | 172 |
|  |  |  |  | **N** | 0.967 | 0.129 | 0.698 | 0.974 | 0.407 | 0.906 | 0.971 | 0.268 | 0.802 | 147 |
|  | **SIMD** | **1** | | | 0.969 | 0.147 | 0.550 | 0.983 | 0.219 | 0.864 | 0.976 | 0.183 | 0.707 | 156 |
|  |  | **2-4** | | | 0.958 | 0.137 | 0.682 | 0.970 | 0.382 | 0.821 | 0.964 | 0.260 | 0.752 | 134 |
|  |  | **5** | | | 0.960 | 0.144 | 0.699 | 0.966 | 0.400 | 0.819 | 0.963 | 0.272 | 0.759 | 144 |
| Activity-level | **Sex** | **Female** | **Y** | | 0.929 | 0.380 | 0.707 | 0.939 | 0.420 | 0.834 | 0.934 | 0.400 | 0.771 | 247 |
|  |  |  | **N** | | 0.960 | 0.336 | 0.689 | 0.947 | 0.404 | 0.874 | 0.953 | 0.370 | 0.781 | 368 |
|  | **SIMD** | **1** | | | 0.950 | 0.282 | 0.824 | 0.958 | 0.333 | 0.913 | 0.954 | 0.308 | 0.869 | 80 |
|  |  | **2-4** | | | 0.941 | 0.107 | 0.695 | 0.940 | 0.430 | 0.919 | 0.941 | 0.269 | 0.807 | 322 |
|  |  | **5** | | | 0.936 | 0.391 | 0.729 | 0.944 | 0.418 | 0.809 | 0.940 | 0.405 | 0.769 | 140 |

**Table S1.** Stratified cross-log conformance checking across sex and socioeconomic deprivation status.

Wave 1 Log conformance to the Wave 2 Petri Net is defined as: Log fitness - $\mathbf{LF}\left( {\mathbf{L}_{\mathbf{1}}\mathbf{, P}}_{\mathbf{2}} \right)$, Precision **-** $\mathbf{P}\left( {\mathbf{L}_{\mathbf{1}}\mathbf{, P}}_{\mathbf{2}} \right)$, generalisation **-** $\mathbf{G}\left( {\mathbf{L}_{\mathbf{1}}\mathbf{, P}}_{\mathbf{2}} \right)$; Wave 2 Log conformance to the Wave 1 Petri Net is defined as: Log fitness - $\mathbf{LF}\left( {\mathbf{L}_{\mathbf{2}}\mathbf{, P}}_{\mathbf{1}} \right)$, Precision **-** $\mathbf{P}\left( {\mathbf{L}_{\mathbf{2}}\mathbf{, P}}_{\mathbf{1}} \right)$, generalisation **-** $\mathbf{G}\left( {\mathbf{L}_{\mathbf{2}}\mathbf{, P}}_{\mathbf{1}} \right)$; $\mathbf{GED}$ – Graph Edit Distance, **SIMD** – Scottish Index of Multiple Deprivation (1 – most deprived areas, 5 – least deprived areas).

## Appendix B.5. Additional conformance checking analysis

From Figure S11, we can infer that the process model captured more similarities in episodes with shorter stays (23.1% fitting traces compared to 6.6% in patients with extended stay). Groups were not aligned in those with and without 1-year mortality (0.3% in positive vs 1.6% in negative group), suggesting high variation in this subset. The remaining subgroups included more fitting traces among complex groups than their negative counterparts. This means that the trace subsets indicated more standardised patterns across both waves for older, multimorbid, intensive therapy and out-of-hours patients.


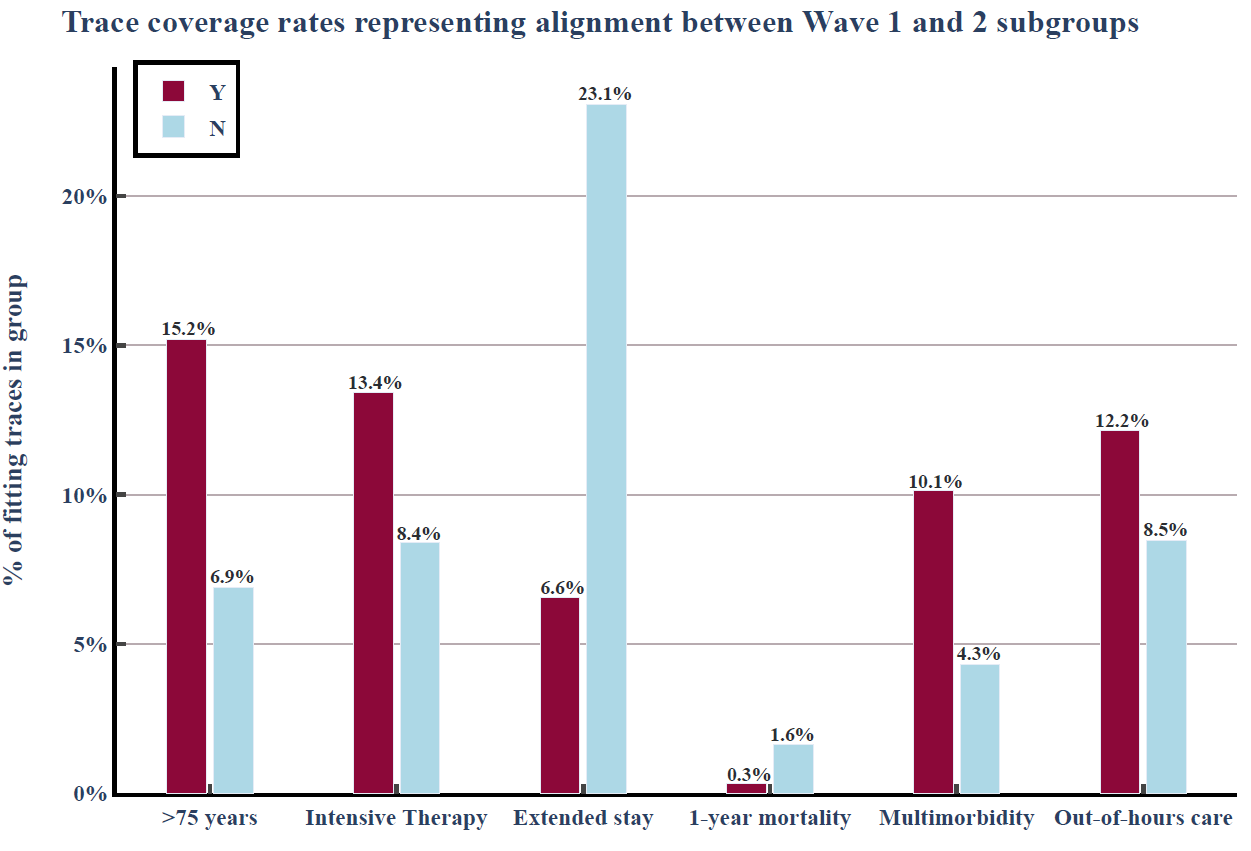


**Figure S11.** Subgroup trace coverage rates on the provider-level, detailing the proportion of fitting traces between the Wave 1 and 2 cohorts at the cross-log conformance checking stage.


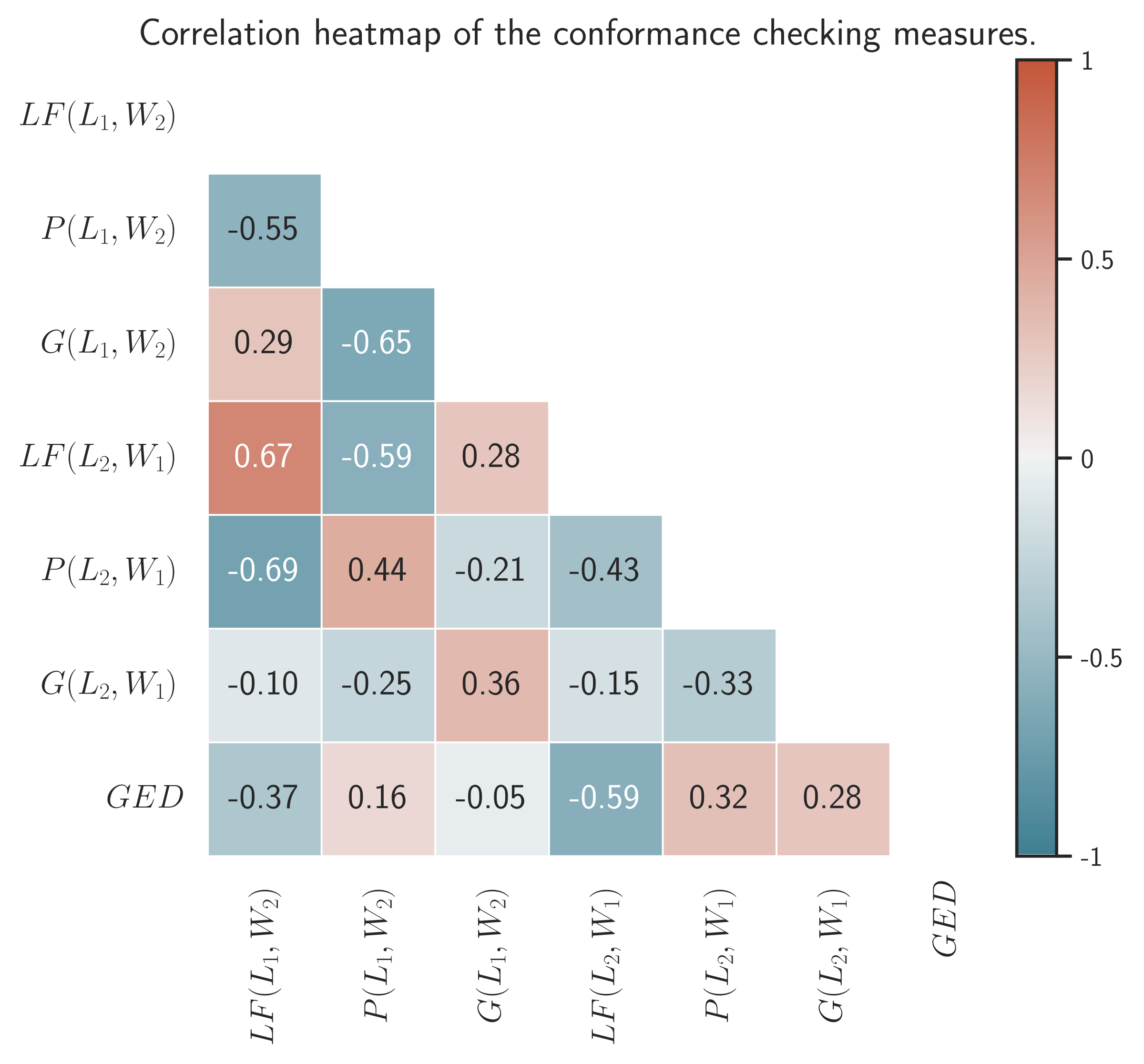


**Figure S12.** Linear correlation heatmap representing the Pearson correlation coefficients between the cross-log conformance checking measures. Wave 1 Log conformance to the Wave 2 Petri Net is defined as: Log fitness - $\mathbf{LF}\left( {\mathbf{L}_{\mathbf{1}}\mathbf{, W}}_{\mathbf{2}} \right)$, Precision **-** $\mathbf{P}\left( {\mathbf{L}_{\mathbf{1}}\mathbf{, W}}_{\mathbf{2}} \right)$, Generalisation **-** $\mathbf{G}\left( {\mathbf{L}_{\mathbf{1}}\mathbf{, W}}_{\mathbf{2}} \right)$; Wave 2 Log conformance to the Wave 1 Petri Net is defined as: Log fitness - $\mathbf{LF}\left( {\mathbf{L}_{\mathbf{2}}\mathbf{, W}}_{\mathbf{1}} \right)$, Precision **-** $\mathbf{P}\left( {\mathbf{L}_{\mathbf{2}}\mathbf{, W}}_{\mathbf{1}} \right)$, Generalisation **-** $\mathbf{G}\left( {\mathbf{L}_{\mathbf{2}}\mathbf{, W}}_{\mathbf{1}} \right)$; $\mathbf{GED}$ – Graph Edit Distance, main measure for graph similarity.

# Supplementary References

1. De Medeiros, A. K. A., Weijters, A. J. M. M. & Van Der Aalst, W. M. P. Genetic process mining: an experimental evaluation. *Data Min Knowl Disc* **14**, 245–304 (2007).

2. Leemans, S. J. J., Fahland, D. & Aalst, W. M. P. van der. Discovering block-structured process models from event logs – A constructive approach.

3. Leemans, S. J. J., Fahland, D. & Van Der Aalst, W. M. P. Discovering Block-Structured Process Models from Event Logs Containing Infrequent Behaviour. in *Business Process Management Workshops* (eds. Lohmann, N., Song, M. & Wohed, P.) vol. 171 66–78 (Springer International Publishing, Cham, 2014).

4. Leemans, S. J. J., Fahland, D. & Van Der Aalst, W. M. P. Scalable Process Discovery with Guarantees. in *Enterprise, Business-Process and Information Systems Modeling* (eds. Gaaloul, K., Schmidt, R., Nurcan, S., Guerreiro, S. & Ma, Q.) vol. 214 85–101 (Springer International Publishing, Cham, 2015).

5. Weijters, A. J. M. M., Aalst, van der, W. M. P. & Alves De Medeiros, A. K. *Process Mining with the HeuristicsMiner Algorithm*. (Technische Universiteit Eindhoven, Eindhoven, 2006).

6. Berti, A. & van der Aalst, W. M. P. A Novel Token-Based Replay Technique to Speed Up Conformance Checking and Process Enhancement. in *Transactions on Petri Nets and Other Models of Concurrency XV* (eds. Koutny, M., Kordon, F. & Pomello, L.) 1–26 (Springer, Berlin, Heidelberg, 2021). doi:10.1007/978-3-662-63079-2_1.

7. Muñoz-Gama, J. & Carmona, J. A Fresh Look at Precision in Process Conformance. in *Business Process Management* (eds. Hull, R., Mendling, J. & Tai, S.) 211–226 (Springer, Berlin, Heidelberg, 2010). doi:10.1007/978-3-642-15618-2_16.
